# Chitosan as a Wound Dressing Starting Material: Antimicrobial Properties and Mode of Action

**DOI:** 10.3390/ijms20235889

**Published:** 2019-11-24

**Authors:** Mariana Adina Matica, Finn Lillelund Aachmann, Anne Tøndervik, Håvard Sletta, Vasile Ostafe

**Affiliations:** 1 Advanced Environmental Research Laboratories, Department of Biology—Chemistry, West University of Timisoara, Oituz 4, 300086 Timisoara, Romania; mariana.matica89@e-uvt.ro; 2 Norwegian Biopolymer Laboratory (NOBIPOL), Department of Biotechnology and Food Sciences, NTNU Norwegian University of Science and Technology, Sem Sælands vei 6/8, 7491 Trondheim, Norway; finn.l.aachmann@ntnu.no; 3 SINTEF Industry, Department of Biotechnology and Nanomedicine, Richard Birkelands veg 3 B, 7034 Trondheim, Norway; Anne.Tondervik@sintef.no (A.T.); Havard.Sletta@sintef.no (H.S.)

**Keywords:** chitosan, antimicrobial properties, mechanism of action, wound dressing, wound healing

## Abstract

Fighting bacterial resistance is one of the concerns in modern days, as antibiotics remain the main resource of bacterial control. Data shows that for every antibiotic developed, there is a microorganism that becomes resistant to it. Natural polymers, as the source of antibacterial agents, offer a new way to fight bacterial infection. The advantage over conventional synthetic antibiotics is that natural antimicrobial agents are biocompatible, non-toxic, and inexpensive. Chitosan is one of the natural polymers that represent a very promising source for the development of antimicrobial agents. In addition, chitosan is biodegradable, non-toxic, and most importantly, promotes wound healing, features that makes it suitable as a starting material for wound dressings. This paper reviews the antimicrobial properties of chitosan and describes the mechanisms of action toward microbial cells as well as the interactions with mammalian cells in terms of wound healing process. Finally, the applications of chitosan as a wound-dressing material are discussed along with the current status of chitosan-based wound dressings existing on the market.

## 1. Introduction

According to the Merriam-Webster Medical Dictionary, an antimicrobial is an agent that either prevents and stops the growth of pathogen microorganisms or even kills them [1].

**Figure 1:**
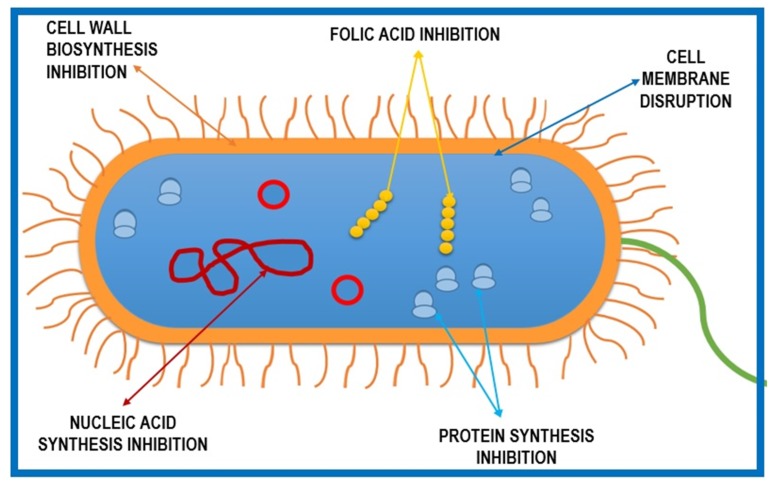
Schematic representation of five basic mechanisms of antibiotic action against microbial cells.

Antimicrobial agents have been used since at least 2000 years ago, when the Ancient Egyptians and Greeks used different plant extracts and mold to treat infections [2]. Since the well-known discovery of penicillin by Alexander Fleming in 1928 through to modern days, antibiotics have been among the most commonly used drugs for treating pathogen infections [3]. Since the number of new classes of antibiotics has decreased and no new classes have been discovered since daptomycin and linezolid in the 1980s, optimizations of already-existing molecules or combinations of different compounds are used for new commercialized products [4]. Figure 1 presents a schematic representation of the mechanisms of action traditionally targeted by antibiotics: (1) disruption of the cell membrane, (2) inhibition of nucleic acid synthesis (DNA/RNA), (3) inhibition of protein synthesis by acting on ribosomes, (4) regulation of enzymes involved in cell wall biosynthesis, and (5) inhibition of folic acid synthesis in the cytoplasm. A modern approach in order to combat antimicrobial resistance would be finding newer targets or using alternative antimicrobials, either natural or synthetic compounds [5,6].

The need for developing new generations of antimicrobial agents is due to the existence of drug-resistant pathogens and the emerging problem of microbial infections. Infections with antibiotic-resistant pathogens are not only difficult to treat but also involve prolonged debility for patients and expensive healthcare [7]. Studies show that more than 70% of chronic wounds are exposed to microbial biofilm infections [8]. A microbial biofilm is described as a well-structured, three-dimensional microbial aggregate that surrounds itself with a self-produced exopolymer matrix containing polysaccharides, proteins, and nucleic acids [9,10,11]. The resistance of biofilm to antibiotic treatments may be explained by various molecular mechanisms [12]. These include, among others, (1) limited diffusion of drugs through the exopolysaccharide matrix [13], (2) the presence of persister cells, which will allow the repopulation of biofilms following antibiotic treatment [14], and (3) possibility of exchanging antibiotic-resistant genes between organisms within the biofilm [12]. Biofilms are by far less susceptible to antibiotics, compared to planktonic cells, and quorum-sensing molecules regulate biofilm formation and expression of specific properties. Also called autoinducers (AIs), quorum-sensing molecules, which regulate most virulence factors, have been identified in various bacterial species. *Staphylococcus aureus* and *Pseudomonas aeruginosa*, two species intensively studied regarding quorum-sensing molecules, are also commonly associated with chronic wound infections [10]. Treating wound infections is more difficult when a biofilm is present. The extracellular polymeric matrix protects the biofilm from inflammatory processes, which are crucial steps in wound healing [15]. The phagocytosis process and complement cascade activation are blocked and the exopolymer matrix represents itself a physical barrier against antimicrobial drug penetration into the wound. Leucocyte penetration and movement are also limited through biofilms; therefore, their ability to produce ROS (reactive oxygen species) is impaired [10].

Besides commonly used antimicrobials such as antibacterials, antifungals, antivirals, and antiparasitics, chemical and natural compounds are some other types of antimicrobial agents that are of great interest not only to the scientific community but also to the public in general, given the fact that antimicrobial resistance is a worldwide threat. In 2018, the European Centre for Disease Prevention and Control (ECDC) stated that nearly 33,000 die every year as a consequence of an infection with antibiotic-resistant bacteria [16]. Therefore, natural and synthetic molecules are an important source for developing new antimicrobials. Antimicrobial polymers are considered the new generation of antimicrobials. Such polymers include, among others, antimicrobial peptides, halogen-containing polymers, phosphor and sulfur derivatives, metal nanoparticles, dendrimers [17], heparin, poly-ε-lysine, gramicidin A, quaternary ammonium polyethyleneimine, and guanylated polymethacrylate, but the most promising is chitosan [18].

Chitosan, a natural cationic polysaccharide consisting of (1→4)-2-amino-2-deoxy-β-d-glucan, is the partially- to fully-deacetylated form of chitin [19]. For many years, there has been great interest in the use of chitin since this biopolymer is the second most abundant after cellulose [20]. There are various sources of chitin: crustacean shells, fungi and algae cell walls, insect exoskeletons, and mollusk radulae [21,22,23,24]. Commercial chitin, (1→4)-2-(acetylamino)-2-deoxy-β-d-glucan, is obtained, following demineralization and deproteinization processes, mainly from crustaceans shells [25,26], which are byproducts of the seafood-processing industry. The seafood-processing industry creates a large quantity of waste every year. About 75% of the total body mass of crustaceans ends up as byproducts (head, tail, backbone, and shell) [27]. Due to a lack of better management of waste, most often, seafood raw materials are discarded back into the sea, burned, or just left out to deteriorate, causing environmental problems [28,29]. Therefore, the extraction of chitin and other valuable substances from crustacean waste is an alternative that can reduce the waste and is an important step in producing useful compounds that have important biological features and applications in various fields. According to The State of World Fisheries and Aquaculture 2018 report by the FAO (Food and Agriculture Organization of the United Nations), global farmed food fish production included approximately 7 million tons of crustaceans [30]. After the edible part of crustaceans has been removed, the remaining material represents an important source of chitin as well as proteins, lipids, pigments, and other molecules [28,31,32,33].

Unlike chitin, chitosan is soluble in acidic aqueous solution due to its acetylation degree. The acetylation degree of chitosan can be expressed either as a percentage of acetylation (DA%) or as the molar fraction of N-acetylated units (DA). Therefore, when the DA is lower than 50% (0.5), dissolution is possible due to the protonation of the amino group at the C-2 position of glucosamine units [34].

The physical-chemical characteristics of chitosan are important in order to understand its functional properties. For example, the degree of acetylation, which refers to the distribution of amino groups along the polymer chain, determines one of the most important features of chitosan, its polycationic nature in acidic media, resulting from the ionization of amino groups. Thus, functional properties of chitosan, such as solubility, swelling ratio, bioactivity, and biodegradation, are influenced by the degree of acetylation. The molecular weight together with the deacetylation degree are the most important physical-chemical characteristics of both chitin and chitosan. By manipulating the molecular weight of chitosan, functional properties can be controlled; for example, viscosity can be reduced and water solubility can be enhanced [35].

Chitosan is also an important tool in research fields due to its significant biological properties such as biocompatibility and biodegradability [36,37,38,39,40], low toxicity [41,42,43,44,45], and antimicrobial [46,47,48,49,50,51,52], antioxidant [49,53,54,55,56], and anti-cancer [57,58,59,60,61,62] properties. Due to all of the valuable features mentioned above, chitosan is widely used in medicine and pharmaceutical fields, the food and agriculture industry, as well as cosmetics and waste treatment [63,64,65,66].

In this review article, we will further discuss chitosan antimicrobial properties and describe its mechanism of action against microbial cells, as well as the interactions with mammalian cells.

## 2. Chitosan Mechanism of Action against Pathogen Microorganisms

Chitosan is one of the most promising natural polymers known to possess antimicrobial properties [67,68]. Interest in the antimicrobial properties of chitosan began many years ago, and some of the first studies that described antimicrobial features of this biopolymer date back to the 1980s and 1990s [69,70]. The main conclusion highlighted by these studies was that chitosan has a bacteriostatic and bactericidal effect, often without making any distinction between these two terms. More recent studies tend to focus more on the bacteriostatic effect of chitosan, although the exact mechanism of action against pathogens is not well known [71,72]. According to the literature, there are a few main generally accepted mechanisms of action of chitosan against microorganisms, which are described below.

### 2.1. Disrupting the Cell Membrane/Cell Wall

Probably the most discussed mechanism refers to the alteration of cell permeability and lysis of the cell membrane. This effect is presumably caused by electrostatic interactions between the positively charged chitosan molecule and negatively charged cell membrane [73,74,75,76,77,78,79,80,81,82,83,84]. The antimicrobial activity of chitosan is based on the presence of the amino groups of the polymer chain. These amino groups can be protonated, giving chitosan a positive charge [78]. Therefore, chitosan becomes soluble in aqueous acidic solutions when the pH value is lower than its pK_a_ value of 6.3, in which case the –NH_2_ groups are converted to a soluble protonated form $-NH_{3}^{+}$ [85,86]. The cell wall of Gram-positive bacteria is composed mainly of a thick peptidoglycan layer containing teichoic acids, which give a negative charge to the bacterial surface, while the Gram-negative bacterial cell wall is strongly negatively charged due to lipopolysaccharides contained in the outer membrane layer. In addition, a similar situation occurs in the case of the fungal membrane and viral envelope, which contain some negatively charged compounds [87]. Fu et al. showed that teichoic acids contained in the cell wall of *Staphylococcus aureus* interact with chitosan derivatives, thus inhibiting the growth of the bacteria, in some cases up to approximately 100% [88]. Li et al. demonstrated that the cell membrane of *Escherichia coli* was damaged by chitosan. They demonstrated extracellular leakage by measuring absorption at 260 nm and 280 nm, estimating, in this way, the amount of DNA and proteins released from the cytoplasm. In addition, electron micrographs obtained using transmission electron microscopy (TEM) revealed clear differences between *E. coli* cells treated with chitosan and those used as a control. Chitosan-treated bacterial cells showed a seriously altered outer cell membrane with cytosolic components coagulated after treatment for 24 h [89]. Vallapa et al. demonstrated that the introduction of additional positive charges to chitosan via heterogeneous quaternization increased the antibacterial activity against both Gram-positive and Gram-negative bacteria, compared with native chitosan [90].

Chitosan may also exhibit antimicrobial activity against fungi. Here, the microorganisms can be divided into two categories: chitosan-sensitive fungi and chitosan-resistant fungi. In this case, the mechanism of action is slightly different from that of bacteria. First, chitosan molecules interact with negatively charged phospholipids, thus affecting the cell membrane and causing the leakage of intracellular material. However, in the case of chitosan-resistant fungi, this polymer seems to be unable to penetrate the cell membrane, and it remains outside the cell surface [91,92]. The fluidity difference of the cell membrane is presumably the reason why chitosan is unable to permeate the cell membrane. The plasma membrane of chitosan-sensitive fungi has more polyunsaturated fatty acids than chitosan-resistant fungi, which indicates that cell membrane disruption by chitosan may depend on the fluidity of membranes [78]. Palma-Guerrero et al. showed that the phospholipid fatty acid composition of the cell membrane is related to chitosan antimicrobial activity. To demonstrate this, they tested the antimicrobial activity of chitosan against a fatty acid desaturase mutant strain of *Neurospora crassa,* which exhibited increased resistance to chitosan compared to the wild type [92].

### 2.2. Interaction with Microbial DNA

Another mechanism consists of the interaction of chitosan hydrolysis products with microbial DNA, thus affecting protein synthesis by inhibiting mRNA [78,93,94]. The ability to penetrate a cell’s membrane and to enter in the cell was observed in the cases of low molecular weight chitosans [72,95,96,97]. Xing et al. investigated the binding of oleoyl-chitosan nanoparticles (OCNPs) to DNA/RNA by examining the effect of OCNPs on the electrophoretic mobility of nucleic acids. The results showed that once the concentration of OCNPs increased, the interactions between bacterial genomes multiplied, and by the time OCNP concentration reached 1000 mg/L, the migration of *E. coli* DNA and RNA was completely inhibited. It is assumed that the negatively charged phosphate groups in the chain of nucleic acids interact with the positively charged amino groups in OCNPs, thus affecting pathogen activity [83]. Galván Márquez et al. suggested that chitosan inhibits protein biosynthesis by testing β-galactosidase expression assays. Their experiments showed that β-galactosidase activity in yeast cells was reduced by 13% when chitosan concentration was 1.25 mg/mL [95].

### 2.3. Chelation of Nutrients by Chitosan

The high chelating capacity of chitosan towards various metal ions (Ni^2+^, Zn^2+^, Co^2+^, Fe^2+^, Cu^2+^) when the pH value is higher than its pK_a_ value could explain the inhibitory effect of chitosan towards microbial growth [19,71,72,98,99,100]. Both Gram-positive and Gram-negative bacterial cell wall components attract divalent metal cations that are beneficial to the microbial cell. The phosphate groups of teichoic acids, contained in the peptidoglycan layer of Gram-positive bacteria, attract especially Mg^2+^ and Ca^2+^ cations, which help to maintain enzymatic functions and the integrity of membranes. Lipopolysaccharides, components of Gram-negative bacterial cell surfaces, give the bacterial cell membrane a negative charge and also have a strong affinity for divalent cations [78]. The lack of these divalent cations makes bacteria more sensitive to chemicals or certain antibacterial agents. Chelating agents, such as ethylenediaminetetraacetic acid (EDTA) or some weak acids, can remove these cations, making the cell wall a more permeable and less effective barrier [101]. It is known that chitosan has chelating properties, so it can chelate metal ions and essential nutrients that are important for the growth of the microbial cell [102,103,104]. At a low pH (below 6.0), the protonated amino groups in the chitosan polymer chain compete with the divalent cations for the phosphate groups found in the lipopolysaccharide or teichoic acid structure. Mansilla et al. showed that the addition of Mg^2+^ and Ca^2+^ to the culture medium increases the positive charge of the membrane and weakens the antimicrobial action of chitosan, proving once again that the bacteriostatic action depends mainly on electrostatic interactions [96].

### 2.4. Formation of a Dense Polymer Film on the Cell Surface

High molecular weight chitosan may deposit and form a dense polymer film on the surface of the cell, preventing nutrient and oxygen uptake, which then inhibits the growth of aerobic bacteria [93,105,106,107]. Champer et al. observed bacterial cells covered with chitosan aggregates using an electron and fluorescence microscope [108]. Due to chitosan deposition, cell walls appeared thicker and covered by an additional layer of vesicular structures. The thickness of the outer membrane prevents the nutrients from entering the cell and also prevents the excretion of metabolic products [78].

## 3. Chitosan Interaction with Prokaryotic Cells

**Figure 2:**
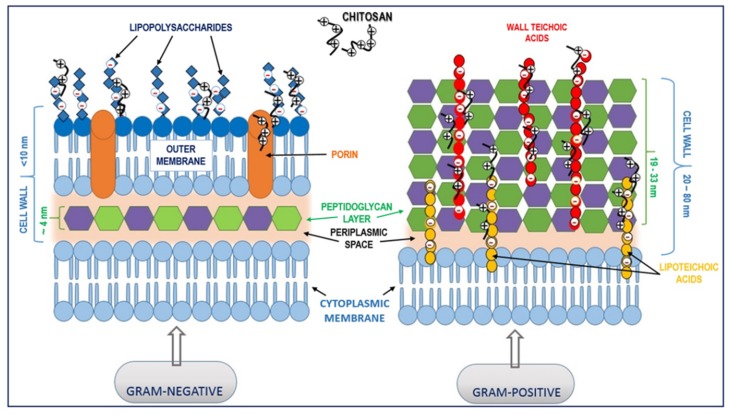
Proposed interactions between chitosan and the bacterial cell wall. Electrostatic interaction between positively charged chitosan molecules and negatively charged lipopolysaccharides (Gram-negative bacteria) and teichoic acids (Gram-positive bacteria) may lead to the blocking of intra/extracellular exchanges or even cell wall disruption and, finally, leakage of cytoplasmic content.

Often, bacteria need to survive even in the most hostile environments; therefore, they developed a specialized cell envelope that is selectively permeable and protects the cell. The antimicrobial effect of chitosan on prokaryotic bacterial cells depends on the electrostatic interactions between this polymer and negatively charged components in the bacterial cell wall (Figure 2).

### 3.1. Interaction with Gram-Positive Bacteria

The difference between Gram-positive and Gram-negative bacteria is in their cell wall structure. When referring to Gram-positive bacteria, the main feature is the presence of teichoic acids, which can interact with chitosan molecules. Teichoic acids are anionic glycopolymers linked to the peptidoglycan layer that, among other functions, play an important role in pathogenesis, modulating susceptibility to cationic molecules. There are two types of teichoic acids: wall teichoic acids and lipoteichoic acid, which are anchored in the bacterial cell membrane. The resistance of Gram-positive bacteria to antimicrobial agents is through teichoic acids and their attached substituents that regulate the negative charge of the bacterial cell, which also prevents the binding of extracellular molecules [109]. This may also be the actual mechanism through which chitosan interacts with the Gram-positive bacterial cell wall. Being positively charged, chitosan can interact with negatively charged teichoic acids, altering cell wall rigidity, disrupting the cell membrane, and eventually entering the cell. Moreover, the presence of d-alanine esters attached to teichoic acids contributes to the bacterial surface charge, and the reduction of d-alanyl content in the cell wall confers susceptibility to glycopeptide antibiotics and some cationic antimicrobial peptides [110]. Studies revealed that the absence of d-alanyl esters can increase the negative charge of the cell surface and facilitate the attraction of cationic molecules, thus increasing the sensitivity of the cell to such antimicrobials [111].

### 3.2. Interaction with Gram-Negative Bacteria

Gram-negative bacteria have a more complex cell surface structure consisting of an outer lipid bilayer structure, the peptidoglycan cell wall, and the plasmatic membrane. The outer lipid bilayer is a distinct feature of Gram-negative bacteria, and its structure contains phospholipids in the inner layer and a single type of glycolipids, known as lipopolysaccharides (LPSs), in the outer layer. The outer layer is a selective barrier with two important features: (1) the selectivity, provided by the porins, which are small protein channels that diffuse certain hydrophilic molecules, and (2) the high negative charge, which is given by LPS molecules. The structure of LPS consists of the lipid A and the inner core, which are the negatively charged groups responsible for the interactions with cationic polymers. Lipid A can also be one possible target for neutralizing the endotoxic effect of LPS [112,113]. Davidova et al.’s study revealed that the interactions occur amongst LPS and the amino groups of chitosan. This was demonstrated using the anionic dye tropaeolin. Experiments showed that at pH value between 4 and 7, where the amino groups of chitosan are ionized, the LPS endotoxins displaced the negatively charged dye molecules from the complex they formed with this cationic polymer [110]. Since porins are known to be responsible for the uptake of nutrients [114], it is possible that chitosan molecules can block the exchange of nutrients by forming a polymer layer on the surface of the cell, thus leading to bacterial cell death.

## 4. Chitosan Interaction with Eukaryotic Cells

### 4.1. Interaction with Fungal Cells

As many studies show, chitosan can inhibit bacterial cell growth and even kill them, but regarding yeast cells, only high concentrations of chitosan can have an antifungal effect and inhibit yeast cells growth [71,87,115,116]. Since chitosan’s cationic nature is the result of the presence of reactive amino groups in its structure, if these groups are blocked, antimycotic activity will be severely reduced [117]. The fungal cell wall is a unique structure, and its components are glycoproteins and polysaccharides, mainly glucan and chitin. These structures are strongly cross-linked to form a resistant assembly that will protect the fungal cell from environmental stress but at the same time will allow the fungal cell to interact with its environment.

Each component of the cell wall is responsible for ensuring cell integrity. For example, if chitin synthesis is perturbed, the fungal cell wall becomes osmotically unstable and deformed. The synthesis of β-1, 3-glucan is necessary for the normal development of fungal cell walls, and the glycoproteins are responsible for maintaining cell shape, intracellular signal transmission, synthesizing other cell wall components, and facilitating molecules absorption, but at the same time protecting the cell against foreign substances [118].

The organization of glycan and proteins at the surface of the cell is unique for each fungal species. The phosphate groups, located in the carbohydrate side chains of highly glycosylated cell wall proteins, give the negative charge at the surface of the fungal cell [119]. Studies have demonstrated the presence of sialic acid on the surface of *Candida albicans* cell walls [118,120]. Anionic groups at the surface of the fungal cell give a high negative charge that could facilitate electrostatic interactions with different molecules and the host cell [121]. As mannoproteins are the major components at the surface of the cell wall and the sialic acids are considered terminal residue in the carbohydrate side chain of mannoproteins [119], electrostatic interactions could occur between chitosan and the fungal cell surface. Soares et al. demonstrated that sialic acids contributed to the electrical negative charge of the cell surface by facilitating the adherence of fungal cells to a cationic substrate (poly-l-lysine) [120].

**Figure 3:**
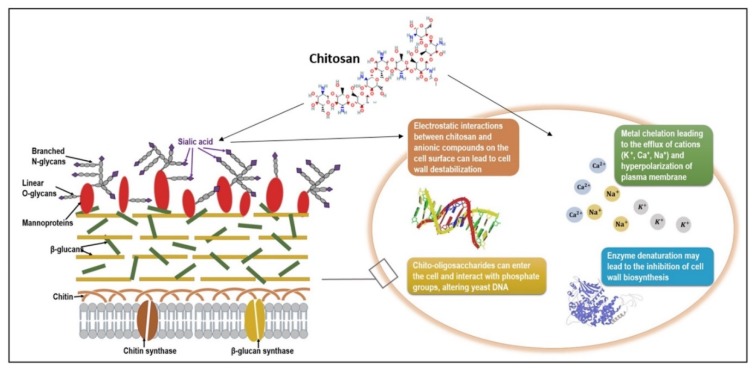
Schematic representation of chitosan mode of action on a *Candida albicans* cell. The fungal cell surface is negatively charged due to the carbohydrate side chains of mannoproteins, mainly sialic acids. Cationic chitosan molecules can cause ionic interactions with anionic groups and destabilize the cell wall. Other mechanisms of action proposed in the literature are metal chelation, enzyme denaturation, and chitosan interaction with phosphate groups of nucleic acids, all causing growth inhibition or even microbial death.

The mechanism by which chitosan inhibits fungal growth is not fully understood yet, but some proposed theories are described below and represented in Figure 3:

(a) Chitosan is a cationic polymer that will interact with the negatively charged cell surface (due to the presence of sialic acid) and can destabilize the electrical charge of the cell wall;

(b) Once the negative charge is decreased, the concentration of the cations at the surface is also affected (mainly K^+^), and the difference between internal and external concentration leads to the efflux of cations;

(c) This efflux of cations will further cause the hyperpolarization of the plasma membrane, therefore causing an increased uptake of Ca^2+^ and the loss of negatively charged molecules (nucleotides, phosphates, substrates for enzyme reaction), affecting the metabolism of the cell and altering important metabolic pathways [122,123].

Moreover, chitosan may form a dense film on the cell surface, which may interfere with fungal growth and activate several defense mechanisms such as chitinase accumulation, callus synthesis, and proteinase inhibitor synthesis and lignification [43]. Another hypothesis, by which yeast cells may recover after long exposure to chitosan or even gain resistance, is the existence of chitinases that may degrade deacetylated chitosan [124,125]. In their study, Oliveira Junior et al. reported that chitosan caused morphological changes and abnormal shapes of fungal mycelia. They tested the effect of chitosan with different molar fractions of acetyl groups (FA 0.16 and 0.18) and degrees of polymerization (DP 1089 and 1242), and both types of chitosan had a direct influence on the three fungal species studied (*Alternaria alternate*—500 μg/mL, *Botrytis cinerea—*1000 μg/mL, *Penicillium expansum—*500 μg/mL). Besides causing a delay in their growth, the micrographs also showed mycelial aggregations, excessive branching, hyphae size reduction, and swelling of the fungal cell wall [109]. Yien et al. concluded that *C. albicans* proved to be more susceptible to chitosan nanoparticles compared with other species [122]. One argument could be the presence of negatively charged sialic acids in the cell wall [120].

### 4.2. Interactions with Mammalian Cells in Wound Healing Processes

Despite the advanced development of antiseptics, infections of wounds remain a major issue. The emergence of multidrug-resistant pathogens leads to a constant need for more efficient topical antimicrobial products. The main drawback is that most of them are highly cytotoxic, affecting the tissue healing process [126].

Apart from antimicrobial and antifungal properties, chitosan is innate, biocompatible, and non-toxic to living cells and tissue. Chitosan biocompatibility has been tested in vitro using different types of cells like fibroblasts, keratinocytes, and hepatocytes, as well as myocardial and endothelial cells [127].

Wounds heal naturally, but there are persons that suffer wound healing disorders, such as diabetic patients who may develop chronic, non-healing wounds that subject the patients to long-term distress and discomfort [113]. In such cases, a long treatment time is required, which will also increase the costs associated with advanced medical care. Therefore, the focus has been on natural therapeutic substances that can accelerate the wound healing process and at the same time be easily accessible to the general population, and chitosan seems to meet all these conditions. Chitosan accelerates the wound healing process by stimulating inflammatory cells, macrophages, and fibroblasts, hence boosting the inflammatory phase. In this way, the inflammatory phase is reduced, and the proliferative phase starts sooner in the wound healing process [128].

Caetano et al. investigated the healing properties of a chitosan–alginate membrane on a cutaneous wound in rats and observed the following: the wound was not infected, fibroplasia was significantly increased, the fibroblasts were arranged better in the newly formed tissue, and the quality of scar tissue was improved. Moreover, on the 7th day of treatment with the chitosan–alginate membrane, the inflammatory infiltrate was significantly reduced, followed by a decrease of neutrophils and CD4+ lymphocytes that might indicate a better regulation of the inflammatory stimulus by the chitosan–alginate membrane. Likewise, the chitosan–alginate complex stimulated the migration of CD11B+ macrophages. These cells are very important as they continue the work of neutrophils, acting as growth factor reservoirs and releasing several enzymes that digest the remaining extracellular content, facilitating the transition to the second phase of the healing process [112]. Chitosan may also have the ability to regulate granulation tissue formation and angiogenesis, assuring the correct deposition of collagen fibers and further enhancing the correct repair of injured dermal tissue [129]. When referring to the wound healing properties of chitosan, the hemostatic and analgesic effect of this biopolymer should be considered. Chitosan’s unique hemostatic properties are independent of the normal clotting cascades [130]. In addition, chitosan can interact with neutrophils and macrophages and regulate the re-epithelialization process and fibroplasia [131,132].

Howling et al. showed that chitosan enhanced fibroblast proliferation, and it seems that the proliferative effect is related to the deacetylation degree of the chitosan. The samples with a high deacetylation degree (84% and 86%) increased the mitogenic activity in fibroblasts, while some samples of chitin had an antiproliferative effect [133]. Possibly, the mechanism by which chitosan enhances fibroblast proliferation is by forming polyelectrolyte complexes with growth factors, heparin, cytokines, or other proteins found at a wound site [134].

At physiological pH, the applications of chitosan are limited due to its poor solubility and the fact that the drug delivery process is less efficient at pH below 6. Therefore, in order to overcome these drawbacks, permanent changes can be introduced into the chitosan backbone. Patrulea at al. developed a water-soluble *O*-carboxymethyl-*N*,*N*,*N*-trimethyl chitosan (CMTMC) with a high degree of substitution (up to 46.6%), which did not exhibit significant toxicity towards human dermal fibroblasts (viability > 80% at 1 and 0.5 mg/mL) [135]. A more recent study published by Patrulea et al. showed that formulations based on carboxylated and trimethylated chitosan (CMTMC) have great potential in wound-healing applications. Foams and hydrogels with a high concentration of RDG (arginyl-glycyl-aspartic acid)-functionalized chitosan (3%) and hyaluronic acid (1.5%) not only showed no toxicity towards human dermal fibroblasts but also promoted proliferation over 7 days and the migration of human dermal fibroblasts [136].

Both hemostatic and analgesic features may be attributed to the positive charge of chitosan. Given the fact that the red blood cells are negatively charged, they can interact with cationic chitosan molecules; therefore, chitosan’s hemostatic effect relies on electrostatic adhesion to blood cells [137]. The exact hemostatic mechanism of chitosan is yet to be fully understood, but theories rely on plasma sorption, erythrocyte coagulation, and platelet activation and aggregation [138]. The protonated amino groups of chitosan attract negatively charged red blood cells, triggering the hemagglutination process [139]. Chitosan stimulates platelet aggregation and adhesion by adsorbing plasma proteins and also signals thrombin, which is a clotting promoter, enhancing the expression of glycoprotein IIb/IIIa (GPIIb/IIIa), a platelet membrane receptor [140]. The analgesic effect of chitosan has been proven since 1984, when Allan et al. reported that chitosan provided a cool and southing effect when applied on open wounds [141]. Huang et al. tested the analgesic effect of chitosan and carboxymethyl chitosan on scalded rats by monitoring the concentration of bradykinin and 5-hydroxytryptophan, potent algogenic substances, using an enzyme-linked immunosorbent assay. Results showed that in the case of carboxymethyl chitosan treatment, the concentrations of bradykinin and 5-hydroxytryptophan were significantly lower than the control group (*p* < 0.05), but in the case of chitosan treatment, the values were same as the control group (*p* > 0.05). Therefore, only carboxymethyl chitosan showed an analgesic effect [142].

Chitosan’s structural characteristics are similar to glycosaminoglycans (GAGs), which are components of the extracellular matrix (ECM); therefore, this feature recommends chitosan use in skin tissue engineering [143]. The immobilization of bioactive molecules on the chitosan matrix, such as cell-adhesive peptides, will stimulate the cell response and cell adhesion process. In this regard, Karakeçili et al. have covalently immobilized mouse epidermal growth factor (EGF) on chitosan membranes. The mouse EGF is responsible for fibroblast proliferation. The results indicated that EGF has enhanced the proliferation of L929 mouse fibroblast cells [144].

Although there are published experimental data on how chitosan interacts with mammalian cells involved in wound healing processes, there is still a lack of knowledge regarding innate sensing of chitosan and the immunological response towards this polymer [145]; as polysaccharides are recognized by the cell receptors and depending on their size and structure, may induce immune responses [146]. The fact that chitin and chitosan are not present in mammalian cells makes them potentially recognizable targets for the innate immune system. However, chitooligosaccharides could be recognized by mammalian chitinases and finally degraded [145]. Another study revealed that chitosan, but not chitin, had triggered inflammatory responses by activating NLRP3 inflammasome in a phagocytosis-dependent manner. While chitosan was a potent NLRP3 inflammasome activator, acetylation of chitosan to chitin resulted in an almost complete loss of activity [147]. Given the fact that chitosan is used in various biomedical applications, research focusing on the immunological response is of great relevance.

## 5. Factors Influencing Chitosan’s Antimicrobial Effect

Many research articles describe the influence that different factors have on the antimicrobial properties of chitosan. Some of the most important factors include molecular weight and degree of deacetylation, pH, type of microorganism, microbial growth media components, the source of the chitosan, and also its derivatives [19].

### 5.1. The Molecular Weight and Degree of Acetylation

**Figure 4:**
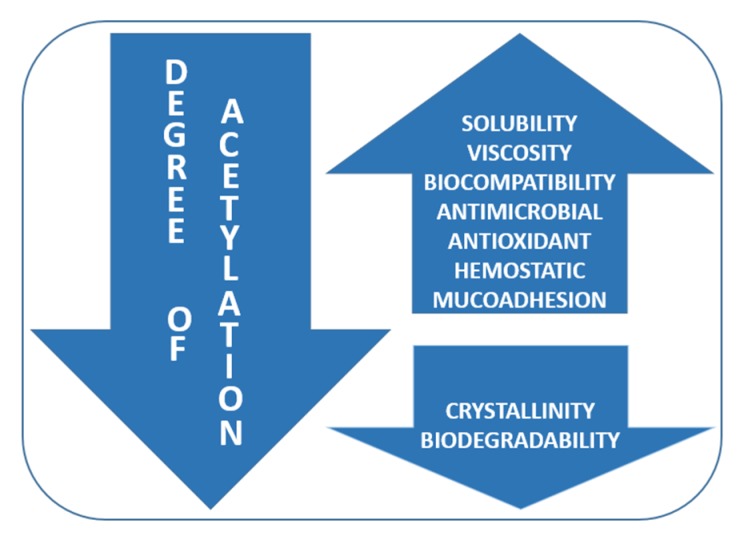
Influence of degree of acetylation (DA) on chitosan physico-chemical and biological properties.

The molecular weight and degree of acetylation of chitosan define several properties of this polymer [148]. The molecular weight of chitosan has a great influence on its biomedical properties. Moreover, the degree of acetylation strongly influences its antimicrobial properties by increasing its solubility and its positive charge. As shown in Figure 4, some physico-chemical properties such as solubility, viscosity, and biocompatibility increase once the DA decreases, while crystallinity and biodegradability are directly proportional to the DA. Biological properties such as antimicrobial, analgesic, antioxidant, hemostatic, and mucoadhesive abilities increase once the degree of acetylation is lower [149].

Chitosans are often classified as high molecular weight (HMW), medium molecular weight (MMW), and low molecular weight (LMW) [19], but the values for each type are not well defined and the literature data is relative and sometimes even confusing (Table 1).

Published data show that the inhibitory mechanism of chitosan against pathogen microorganisms is also related to its molecular weight. Thus, while HMW chitosan forms a dense layer on the surface of the cell, preventing nutrient uptake [89], the much smaller chitosan molecules seem to penetrate the membrane and even bind to DNA and RNA [98,159]. The antibacterial effect is dependent on both molecular weight (MW) and type of microorganism. Tin et al. tested the antimicrobial activity of four different MW chitosan samples (LMW, MMW, HMW, and chitosan oligosaccharide lactate) against *Pseudomonas aeruginosa*. The results showed that the MIC (minimum inhibitory concentration) values were higher for chitooligosaccharides (4096 µg/mL) compared to other chitosan samples (32 µg/mL) [160]. Another study performed by Batista et al. illustrates the importance of MW on antimicrobial activity. They have tested two types of chitosan (HMW and LMW) against *E. coli* (Gram-negative), *L. casei*, and multiresistant strain *S. aureus* (both Gram-positive). Only MMW chitosan was antibacterially active, and only against *E. coli* and *L. casei*, while *S. aureus* was resistant to both chitosan samples [161]. As shown in Table 2, many other studies reported similar data regarding the MW of chitosan and chitosan derivatives.

### 5.2. pH Effect

The beneficial properties of this polymer depend strongly on its solubility, in either water or other commonly used solvents. In its crystalline form, chitosan is soluble in aqueous solutions only at a pH value under 7. Its solubility is due to the protonation of its amino groups under acidic conditions. The main disadvantage of chitosan is its low solubility, and over the past years, there has been a growing interest in the chemical modification of this polymer in order to improve its solubility [149,165,166]. At a pH value of approximately 6.0, chitosan carries a positive charge, being able to interact electrostatically with various negatively charged molecules. Therefore, the antimicrobial mechanism of chitosan is pH-dependent.

As described earlier in this paper, chitosan interacts with the negatively charged cell membranes of bacteria and fungi, leading eventually to the death of the microbial cell [167]. Many studies show that chitosan’s antimicrobial activity is minimal at higher pH conditions. Younes et al. demonstrated that when reducing pH values, the adsorption on bacterial cell surfaces will increase, probably due to the highly positive charge on the chitosan polymer chain [34]. On the contrary, at higher pH (above 6), chitosan will lose its charge, and due to the deprotonation of its amino groups, it will precipitate from solution. Devlieghere et al. observed during their study that native chitosan showed greater antifungal activity against *Candida lambica* at a pH value of 4.0 rather than 6.0 [106]. Helander et al. also observed a greater antimicrobial effect of chitosan at lower pH conditions, while the inhibitory activity decreased with increasing pH [77]. Erdem et al. tested the influence of pH on the antimicrobial activity of chitosan against two strains: *S. aureus* and *Aeromonas hydrophila*. The pH values selected were 5.0, 6.0, 7.0, and 8.0. Chitosan showed an increased inhibitory effect at lower pH (5–6) against both strains, reducing the viable cells from their initial populations to 1.80–0.57 log CFU/mL for *A. hydrophila* and 1.70–1.00 log CFU/mL in the case of *S. aureus*. In addition, they observed that unmodified chitosan does not present antimicrobial activity at a pH of around 7.0 [168].

The results of Batista et al. are similar in that they observed the highest inhibitory effect of chitosan against *E. coli* and *Lactobacillus casei* at a pH value of 5.7 (*L. casei*) and 5.5 (*E. coli*) [161]. While testing the antifungal activity of LMW chitosan against several species of clinical isolates of *Candida* sp., Alburquenque et al. observed an increased inhibitory effect at a pH value of 4.0, with the MIC being up to 4 times lower than at neutral pH. Their theory was also based on the fact that the increased antifungal activity is due to the protonation of the amino group of chitosan units at pH 4.0, as the pK_a_ of LMW chitosan is 6.3 [169].

### 5.3. Source of Chitosan

Researchers have already investigated and compared the antimicrobial effect of chitosan extracted from different sources. Tajdini et al. compared the antimicrobial effect of shrimp chitosan with fungal chitosan against twelve bacterial and fungal species. The results showed that fungal chitosan was more efficient against *P. aeruginosa*, *E. coli*, *Candida glabrata*, and *C. albicans* in comparison to the shrimp chitosan, the MIC values being twice smaller for fungal chitosan. Against the other microorganisms tested, the antimicrobial effect was similar, and MIC values were the same for both types of chitosan, indicating that fungal chitosan can be an effective and suitable alternative to shrimp chitosan [170]. Chien et al.’s study revealed that chitosan extracted from shiitake mushrooms was more effective than shrimp chitosan against some of the pathogenic microorganisms tested [98]. Tayel et al. published a study that showed that chitosan extracted from *Mucor rouxii* DSM-1191 exhibited pronounced antifungal activity against different *Candida albicans* strains. Four types of fungal chitosan were tested, and the most active against the fungal strain had the highest deacetylation degree (94%) and the lowest molecular weight (32 kDa). [162]. Moussa et al. also demonstrated that fungal chitosan (deacetylation degree of 89.2% and a molecular weight of 2.4 × 10^4^ Da) extracted from *Aspergillus niger* mycelia had a high antimicrobial effect against two foodborne pathogens (*Salmonella typhimurium* and *Staphylococcus aureus*) [171].

Extraction of chitosan from fungal sources has many advantages over crustacean sources:

(a) Eliminates the harsh chemical processes that may cause environmental pollution;

(b) The raw material can be available throughout the entire year, and there are no risks of heavy metal contamination;

(c) The demineralization step is no longer necessary for the extraction of chitosan from fungal mycelia;

(d) Different types of waste products can be used to grow fungal cultures, reducing marine pollution by the removal of nitrogen, proteins, and other compounds from waste [107,117].

Moreover, as an alternative to crustacean-derived chitosan, fungal strains may be adapted to industrial needs by applying genetic modifications in order to produce larger amounts of chitin for further production of higher-quality chitosan [172].

However, some important advantages of chitosan extraction from crustacean waste are worth mentioning. First, crustacean waste is an important source of pollution; therefore, obtaining chitosan from this waste material will give value-added products. Furthermore, if enzymatic extraction methods are used, the harsh environmental impact that chemical extraction methods involve will be reduced, and the result will be a well-defined chitosan, obtained by a controlled, non-degradable process [173,174].

### 5.4. Type of Microorganism

It is a fact that chitosan possesses innate antimicrobial properties, and most studies show that its antimicrobial properties depend on the type of microorganisms. Although most of the results indicate that chitosan has a more pronounced antimicrobial effect on Gram-positive bacteria rather than Gram-negative [175], the literature is contradictory, as other studies have demonstrated the opposite [124,176]. For example, Fernandes et al. showed that the antimicrobial effect of chitosan was dependent on the target microorganism as well as the molecular weight. Thus, chitooligosaccharides had a higher antibacterial effect on Gram-negative species (*E. coli*, *Klebsiella pneumoniae*, *P aeruginosa*, MIC values of 0.10%, 0.10%, 0.20%, respectively) compared with Gram-positive ones (*S. aureus* and *S. epidermidis*, MIC values of 0.25% and 0.20%, respectively). In the case of Gram-positive bacteria, high-molecular-weight chitosan exhibited a stronger antibacterial effect than chitooligosaccharides (MIC values of 0.10% for both strains), while for Gram-negative bacteria, MIC values were higher (0.25% for *E. coli* and *K. pneumoniae* and 0.5% for *P. aeruginosa*). Likewise, for *C. albicans*, the results were rather similar to those detected for Gram-positive bacteria; chitooligosaccharides inhibited fungal growth at an MIC value of 0.25%, while the MIC value for high-molecular chitosan was 0.15% [177].

The result of a study by Chung et al. showed that chitosan’s inhibitory effect was higher against Gram-negative bacteria. Their hypothesis was related to the hydrophilicity of the bacterial cell wall. The results obtained showed a greater hydrophilicity in Gram-negative bacterial cell walls then Gram-positive ones; therefore, more negatively charged bacterial surfaces had greater interaction with positively charged chitosan [178].

A recent study on the antimicrobial effect of chitosan against bacterial contamination on the surface on Iranian banknotes shows that the inhibitory effect is stronger against Gram-positive bacteria, and a possibility of avoiding bacterial contamination through banknotes could be their coating with chitosan [179].

The components found in the growth media used to cultivate the microorganisms may also influence the antimicrobial properties of chitosan. Devlieghere et al. demonstrated that media food components could influence the effect of chitosan on microbial growth. For example, 0.005% chitosan was enough to inhibit *Candida lambica* growth, and small amounts of starch (1% *w*/*v*) did not affect chitosan antimicrobial properties. However, high amounts of starch (30% *w*/*v*) significantly decreased the activity of chitosan against *C. lambica*. In addition, only 1% of NaCl was enough to inhibit the antimicrobial activity of chitosan [106].

### 5.5. Chitosan Complexes

The main purpose of producing chitosan complexes with other compounds is to improve its beneficial properties, most of all, its antibacterial effects, and accelerate wound healing.

Various studies present different complexes between chitosan and other natural or synthetic compounds in order to improve its antimicrobial effect and accelerate the wound healing process. For example, Liu et al. developed an antibacterial material based on chitosan oligosaccharide-N-chlorokojic acid mannich base (COS-N-MB). The resulting COS-N-MB complex with synergistic antibacterial effects showed excellent inhibitory activity against pathogenic bacteria. The mechanism of action seems to rely on the adsorption to bacterial cell walls through electrostatic interactions and chelating metal ions [125]. Biswas et al. developed two distinct wound-dressing materials by loading porous chitosan/poly(vinyl alcohol) (CS/PVA) scaffolds with Ag and Se using an in situ deposition method. The Ag-CS complex showed significant inhibition of bacterial growth and reduction of cytotoxicity to fibroblasts, while the Se-CS complex damaged bacterial cell membranes and showed no toxicity towards fibroblasts [180]. Ma et al. observed that the incorporation of glycerol enhances mechanical properties and maintains the good water vapor permeability and wettability of chitosan-based membranes [181]. The study of Kaygusuz et al. demonstrated that cerium ion–chitosan crosslinked alginate films show antibacterial activity against both Gram-negative (*E. coli*) and Gram-positive bacteria (*S. aureus*) and also show high mechanic resistance, flexibility, and UV protection [182]. Anjum et al. developed antimicrobial and scar-preventive wound dressings by blending chitosan (CS), polyethylene glycol (PEG), and polyvinyl pyrolidone (PVP) on a cotton fabric. The cotton fabric was used as a support layer for the CS-PEG/PVP gel; PEG increased the mechanical properties, and polyvinyl pyrolidone (PVP) helped hydrogel formation [183]. Wahid et al. prepared Carboxymethyl chitosan (CMCh) supramolecular hydrogels cross-linked by metal ions (Ag^+^, Cu^2+^, and Zn^2+^) that showed moldability, elasticity, high mechanical properties, and a rapid and facile gelation process (within seconds). Moreover, the metal ions added to the CMCh membranes significantly enhanced antimicrobial activity (Zn-loaded CMCh membranes had higher efficiency against all bacteria tested) [184].

Although chitosan’s antibacterial activity is lower compared with conventional antibiotics, its unique chemical behavior, namely the presence of three reactive functional groups (an amino group at the C-2 position, a primary OH group at the C-6 position, and secondary OH groups at the C-3 position), make this polymer available for further chemical modification in order to improve its antimicrobial activity [72,125].

## 6. Chitosan Antimicrobial Wound Dressings

Wound infections can severely affect the healing process, and if not properly treated, they can lead to sepsis and even to patient death [185]. The human skin acts as a barrier against external factors, but when injured, it loses its efficacy and becomes vulnerable to pathogenic infections [186,187].

The ideal wound dressing (Table 3) should meet some requirements such as:

(1) representing a physical barrier that is permeable to oxygen but at the same time maintains or provides a moist environment; (2) sterile and non-toxic and protective against microorganism infections; (3) providing an appropriate tissue temperature to favor epidermal migration and promote angiogenesis; (4) non-adherent to prevent traumatic removal after healing [188]. All the above-mentioned properties are characteristic of an ideal wound dressing, but it is difficult for one type of dressing the meet all these requirements.

In addition, it is preferable that wound dressings contribute to skin regeneration. In this regard, a variety of wound-dressing materials have been developed either based on synthetic or natural polymers. As seen in Table 4, there are many major types of wound-dressing materials: fibers, gels, membranes, films, sponges, and hydrocolloids. Although the terms “film” and “membranes” could be use to describe the same type of wound dressing, referring to a device developed to isolate a surface from its environment, we have chosen to use and describe the terms separately, mainly due to the fact that the terms vary among authors [195,196,197,198,199]. Moreover, both terms are used accordingly by experts from different fields; for example, the term “membrane” is employed mainly in the biological and health sciences, while professionals from engineering science use the term “film”. The distinction between the two terms can be made based on the moisture content, as membranes are hydrate films while films are considered dried membranes [200].

The most suitable antimicrobial polymer should be easy and inexpensive to synthesize, stable for long-term usage, biodegradable and non-toxic, and most importantly, have a biocidal effect to a broad spectrum of pathogens [207]. Chitosan, as a natural cationic polysaccharide, seems to possess all of the above-mentioned properties [128]. Moreover, the difference between chitosan-based products and traditional dressings like gauze or cotton wool is that chitosan participates actively in wound healing processes [188].

Chitosan derivatives have become important tools in many different areas such as water purification [208,209,210,211], food and paper industries [212,213,214,215], and most importantly, medicine and pharmacy [127,135,216,217]. Allan et al. were amongst the first to propose the antimicrobial activity of chitosan [218], and since then, the idea of developing chitosan derivatives has become more and more pronounced amongst scientists. In Table 5, the effects of different types of chitosan on some pathogenic microorganisms are presented. Although we cannot compare the results obtained in different experimental studies, within the same set of experiments, we can evaluate the effect of chitosan on different species of microorganisms. Because human health can be severely impaired by pathogenic microorganisms, the search for more efficient antimicrobial agents is even more imperative.

### 6.1. Chitosan Fibers

There are several common ways to produce chitosan fibers, the first published data being reported in 1926 [90]. Fibers can be produced by a dry and wet spinning method using different solvents. A more recent technique used by modern-day scientists is electrospinning (ESP). The main advantage of this method is that it allows the fabrication of polymer nano- and microfibers, depending on the processing conditions [228]. Porous fiber membranes have a large potential in wound-healing applications due to their high porosity and the ability to mimic the skin’s extracellular matrix.

Zhou et al. synthesized *N*,*N*,*N*-trimethyl chitosan (TMC) fibers with different degrees of quaternization (DQ 19%, 25%, and 32%). TMC fibers showed higher antibacterial activity against Gram-negative *E. coli* (>63%) and Gram-positive *S. aureus* (>99%) than chitosan fibers (24%). In addition, TMC fibers showed no cytotoxicity toward mouse fibroblasts and the TMC DQ of 25% significantly increased wound reepithelization compared to the control (chitosan fibers). The results indicated that a high DQ increased antibacterial activity due to the quaternary ammonium groups with permanent positive charges. The cationic charge is the key to the binding process of the polymer to the negatively charged bacterial cell membrane [229].

### 6.2. Chitosan Hydrogels

Hydrogels are 3D polymer networks that can absorb large amounts of water and are moist, flexible, and soft materials with a wide range of applications in biomedical fields. The high content of water and the mechanical properties of hydrogels make them compatible with most living tissues, and they improve the healing process [127]. The hydrophilic gel keeps the wound bed moist, providing a non-adherent environment, and their swelling capacity helps with wound exudate absorption [230]. Another advantage of hydrogels is that they can follow the geometry of the wound, assuring a good superficial contact, which can be very helpful even in third-degree burns [165].

In addition to their main purpose (drug and/or growth factor delivery while speeding up the healing process), hydrogels must also prevent the microbial infections that can develop in this moist environment. Antimicrobials can be either covalently bound to the hydrogel network, noncovalently encapsulated in hydrogels, or the hydrogel can possess inherent antimicrobial properties. Amongst other antimicrobial polymer-based gels, chitosan-based hydrogels are of great interest for the scientists [231].

### 6.3. Chitosan Membranes

Amongst the variety of wound dressings available, the porous membrane dressings are considered to be the ones that meet most of the requirements of an ideal wound dressing [51]. However, the moist environment provided by membranes also facilitates pathogen proliferation, thus it is highly necessary to impregnate the membranes with an antimicrobial agent.

The simplest method of developing chitosan membranes is a solution casting–evaporation technique, by which chitosan is first solubilized in acetic acid solution [232]. The main drawback of this method is that acetic acid, along with chemical cross-linkers, such as carbodiimide or glutaraldehyde, among others, possess cytotoxic effect on mammalian cells; therefore, this is a major limitation to wound healing. To overcome these disadvantages, Ma et al. developed chitosan–glycerol membranes loaded with tetracycline hydrochloride (TH) and silver sulfadiazine (AgSD) through a simple solution casting–evaporation method and evaluated the bacterial growth inhibition against *E. coli* and *S. aureus* by measuring the inhibition zone diameter. The difference was that they used chitosan floccule suspension instead of an acetic acid solution. Thus, chitosan–glycerol membranes loaded with antibacterial agents not only inhibited bacterial growth in a significant manner, but glycerol also enhanced the membranes’ mechanical properties, promoting wound healing [233].

### 6.4. Chitosan Films

Güneş et al. developed a chitosan-based film incorporating *Hypericum perforatum* oil that proved to have an antimicrobial effect on *E. coli* and *S. aureus*. The results showed that the antimicrobial effect increases with the concentration of essential oil incorporated into the chitosan matrix. Thus, the diameter of the zone of inhibition of simple chitosan films against *E. coli* was 2 cm compared to 2.9 cm in the case of chitosan films incorporated with 1.5% *H. perforatum* oil. *S. aureus* proved to be more resistant than *E. coli*, with a diameter of the inhibition zone of chitosan films with an essential oil concentration of 1.5% equal to 1.97 cm [223].

There are numerous chitosan-based products on the market, and one example is the HemCon^®^ dressing, which is a chitosan acetate bandage that was designed as a hemostatic dressing with antimicrobial properties. Burkatovskaya et al. investigated its effect on infected and non-infected excisional wounds in mice. The wounds infected with Gram-negative species (*P. aeruginosa* and *Proteus mirabilis*) and left untreated led to serious infections that caused the death of mice, but the chitosan acetate bandage applied on the infected area rapidly killed bacteria [126].

### 6.5. Chitosan Sponges

Sponges are foam-like, solid structures that can absorb large amounts of liquid due to their high porosity. Besides possessing antimicrobial properties, chitosan sponges are widely used as wound dressings, being able to absorb wound exudates and also enhancing tissue regeneration [165].

Anisha et al. developed an antimicrobial chitosan–hyaluronic acid/nanosilver composite sponge for the treatment of infected diabetic wounds. The sponges showed an antibacterial effect against the major bacterial strains known to infect a wound (*E. coli*, *S. aureus*, *P. aeruginosa*, *K. pneumoniae*, and even MRSA—Methicillin-resistant *Staphylococcus aureus*). However, cytotoxicity tests showed that higher concentrations of nanosilver in the sponges decreased cell viability [234]. Another study published by Shao et al. showed that silver-sulfadiazine-loaded chitosan composite sponges had high porosity and excellent swelling behaviors and possessed a broad spectrum of antibacterial activity (*E. coli*, *C. albicans*, *S. aureus*, and *B. subtilis*). The cell viability results performed by 3-(4,5-Dimethyl-2-thiazolyl)-2,5-diphenyl-2H-tetrazolium bromide (MTT) viability assay and fluorescence staining method on HEK293 cell lines indicated that the sponges had no significant cytotoxicity; therefore, CS/AgSD composite sponges have potential applications as antimicrobial wound-dressing materials [235]. Pei et al. fabricated AgNP-loaded silk fibroin (SF)/carboxymethyl chitosan (CMC) composite sponges that showed effective antibacterial activity against *S. aureus* and *P. aeruginosa.* The addition of CMC increased the water vapor transmission rate and improved the water absorption capacity and retention ability of the sponge, all of these being important properties of a wound dressing [236]. Mi and team developed a sponge-like asymmetric chitosan membrane by an immersion–precipitation phase-inversion method. The sponge-like membranes showed many advantages a wound dressing requires, such as excellent oxygen permeability, and controlled evaporative water loss, promoted fluid drainage ability, and inhibited exogenous microorganism invasion due to the inherent antimicrobial properties of chitosan. Moreover, histological tests on rat wounds showed an increased epithelialization rate and well-organized deposition of collagen in the dermis [237].

### 6.6. Chitosan Hydrocolloids

The composition of a hydrocolloid consists of an external layer of polyurethane and one internal layer composed of hydrophilic colloidal particles (carboxymethylcellulose, gelatin, pectin). A hydrocolloid dressing will absorb wound exudate due to its internal layer of gelatin, pectin, and carboxymethylcellulose. The external polyurethane layer will seal the wound, not only enabling gas exchange and preventing external contamination of the wound, but also maintaining an acidic pH at the wound bed, facilitating autolytic debridement [90,204]. In addition, they promote angiogenesis, increase the number of fibroblasts and the number of collagen fibers, and enhance the production of granulation tissue, all of each are very important steps in the healing process [204].

Hiranpattanakul et al. developed a chitin/chitosan hydrocolloid (CCH) wound dressing, and properties like their water absorption, enzymatic degradation, and antibacterial activity against Gram-positive and Gram-negative bacteria were evaluated, as well as their biocompatibility with the L929 cell line. The CCH showed antibacterial activity against *E. coli* and *S. aureus* and showed no cytotoxicity against the L929 fibroblasts [231]. Yanagibayashi et al. developed another functionalized wound dressing to stimulate wound healing in diabetic mice. The hydrocolloid composed of alginate, chitin/chitosan, and fucoidan (ACF-HS) had important properties like adherence, ease of application and removal, providing a moist environment, and absorbing exudate. Moreover, the histological examinations of the wounds showed advanced tissue and capillary formations, starting on the 4th day of treatment [238].

The multitude of wound dressings already existing on the market, from sponges to granules, gels, and sprays, only prove that chitosan is a promising polymer for wound-healing applications, being versatile and suitable for many types of wounds (Table 6). Although the majority of chitosan-based wound dressings, presented below, are hemostatic dressings developed to stop moderate to severe bleeding, they also have specific features that direct them to certain types of wounds. For example, Chitoderm^®^ plus is a wound dressing based on a strong non-adhesive superabsorber, with chitosan coating, that has bacteriostatic action and actively accelerates healing processes. The manufacturer recommends it for numerous types of wounds, from acute to chronic, exudating, contaminated wounds, venous leg ulcers, diabetic, and first- and second-degree burns [239]. Another product, Celox™, is a hemostatic chitosan-based wound dressing that clots hypothermic blood and blood treated with blood-thinning drugs, can be easily removed from the wound site, and the residual material is naturally absorbed by the body [240]. The manufacturers of Opticell gelling fibers had an interesting approach in developing this chitosan-based dressing. Not only can the dressing be worn up to seven days, but the chitosan-based Cytoform technology allows the absorbent fibers within the dressing to transform into a gel. Indicated for the control of minor bleeding, the gelling action helps manage drainage and the removal of dead, damaged tissue, trapping it until the later removal of the dressing [241]. A similar approach is seen for the LQD spray dressing, which contains chitosan as active ingredient, that has been modified to have the highest degree of deacetylation of any chitosan product and a much higher positive charge. The LQD spray is applied as a spray and within two minutes of application, it forms a chitosan membrane over the wound. This dressing, which is indicated for the external treatment of chronic and acute wounds and superficial and partial thickness burns, providing physical protection and suppressing the secretion of proinflamatory cytokines, contributing to pain relief, has a hemostatic and antibacterial effect [242].

The current regulatory status of chitosan given by the US FDA (United States Food and Drug Administration) is defined as GRAS (generally recognized as safe) for wound-dressing applications [243,244,245] and also tissue engineering [246,247]. Moreover, chitosan has been approved as a dietary supplement in Japan, Italy, and Finland [248]. Therefore, there is a growing number of chitosan wound dressings that meet the requirements of various types of wounds, and due to the advantages provided by this polymer, the use of chitosan as a wound-dressing material will only be increasing in the future.

## 7. Conclusions

There is no doubt that chitosan possesses important features that can be very helpful in solving modern-day challenges regarding various areas of interest such as biomedicine, pharmaceutical, cosmetic, and food industries, wastewater treatment, and agricultural pest management.

Chitosan may be one of the most important biopolymers that can be used to produce wound dressings that not only accelerate the wound healing process but also prevent infection of the wound, a necessary step in the wound healing process.

The mechanism of action against microbial cells is not entirely defined, but numerous studies have presented similar results; therefore, researchers have formulated some hypotheses by which chitosan can inhibit microbial growth:

(1) electrostatic interactions occur between cationic chitosan and anionic molecules at the microbial cell surface, which may lead to cell wall disruption and intracellular components leakage;

(2) LMW chitosan can penetrate the cell membrane and interact with DNA, thereby interfering with protein synthesis processes;

(3) Another mechanism of action for chitosan would be the chelation of nutrients and essential metals that are fundamental to cell stability.

Used as a wound dressing, chitosan stimulates the natural healing process and has no toxicity to the mammalian cell. Being biocompatible, chitosan has a known metabolic pathway. Once it enters the human body, it can be hydrolyzed by enzymes, such as lysozyme, present in mucus, tears, and saliva, to an important sugar, glucosamine, that it is already present in the human body.

The existence of numerous chitosan-based wound dressings on the market shows the importance of this biopolymer in accelerating the wound healing process, and we may consider chitosan as a cost-effective solution not only for the treatment of acute wounds but also in the case of severe chronic wounds, such as diabetic ulcers.

## Figures and Tables

**Table 1:** Molecular weight of different types of chitosan.

Type of Chitosan	Molecular Weight Range	Reference
Oligochitosan	4.7 kDa	[150]
Low molecular weight chitosan (LMW)	~10 kDa	[151]
	22 kDa	[152]
	120 kDa	[153]
	13 kDa	[154]
	4.8 kDa	[155]
	3.69 kDa	[156]
	50–90 kDa	[157]
	50–190 kDa	[158]
Medium molecular weight chitosan (MMW)	250 kDa	[153]
	190–310 kDa	[158]
	190–310 kDa	[157]
High molecular weight chitosan (HMW)	64.8–170 kDa	[151]
	340 kDa	[153]
	310–375 kDa	[157]

**Table 2:** Minimum inhibitory concentration (MIC) of different types of chitosan (C).

Microorganism	Type of Cs	MIC	References
*P. aeruginosa*	LMW Cs	32 μg/mL	[160]
	MMW Cs		
	HMW Cs		
	Cs oligosaccharide lactate	4096 μg/mL	
*Candida albicans*	Cs 32 kDa	2 mg/mL	[162]
	Cs 38 kDa	2.3 mg/mL	
	Cs 138 kDa	2.5 mg/mL	
	Cs 184 kDa	3.3 mg/mL	
*Streptococcus mutans*	LMW Cs	2.5 mg/mL	[163]
	Nano Cs	1.25 mg/mL	
*Streptococcus sobrinus*	LMW Cs	2.5 mg/mL	
	Nano Cs		
*Streptococcus sanguis*	LMW Cs	1.25 mg/mL	
	Nano Cs		
*Streptococcus salivarius*	LMW Cs	1.25 mg/mL	
	Nano Cs	2.5 mg/mL	
*Pseudomonas aeruginosa*	Cs 322.04 kDa	60 μg/mL	[164]
*Staphylococcus aureus*		80 μg/mL	
*Streptococcus* sp.		60 μg/mL	
*Streptococcus pneumoniae*		100 μg/mL	
*Escherichia coli*		80 μg/mL	
*Staphylococcus aureus*	Cs 41.1 kDa	32 μg/mL	[53]
	Cs 14.3 kDa	32 μg/mL	
	Cs 5.06 kDa	32 μg/mL	
*Escherichia coli*	Cs 41.1 kDa	64 μg/mL	
	Cs 14.3 kDa	32 μg/mL	
	Cs 5.06 kDa	16 μg/mL	

**Table 3:** Properties of an ideal wound dressing.

Characteristics of Wound Dressing	Importance in Wound Healing	Reference
Providing a moist wound environment	Prevents dehydration and cell death Promotes epidermal migration and angiogenesis Maintains moisture at the wound bed	[189]
Removal of excess exudate	Exudate is essential for the wound healing process, but excess exudate can cause healthy tissue maceration, resulting in a chronic wound.	[190]
Allows gaseous exchange	Oxygenation controls exudate levels and stimulates epithelialization and fibroblasts.	[191]
Prevents infections	Microbial infections delay the wound healing process by prolonging the inflammatory phase and by inhibiting epidermal migration and collagen synthesis.	[192]
Low adherence and painless removal	Removal of adherent dressing can be painful and can cause further damage to granulation tissue.	[193]
Cost-effective	An ideal dressing should assure the wound healing process at a reasonable cost.	[194]

**Table 4:** Advantages and disadvantages of major wound dressings.

Wound Dressing Type	Advantages	Disadvantages	Reference
Sponges	high porosity thermal insulation sustain a moist environment absorb wound exudates enhance tissue regeneration	mechanically weak may provoke skin maceration unsuitable for third-degree burn treatment or wounds with dry eschar	[201,202]
Hydrogels	high absorption properties provide a moist environment at the wound site water retention oxygen permeability ensure the solubility of growth factor/antimicrobial agents	weak mechanical properties need for a secondary dressing	[127,135,203]
Hydrocolloids	non-adherent high density painless removal high absorption properties	can be cytotoxic have an unpleasant odor low mechanical stability maintain acidic pH at the wound site	[90,204]
Films	impermeable to bacteria allows the healing process to be monitored painless removal	hard to handle non-absorbent adhere to the wound bed and cause exudate accumulation	[48,188]
Membranes	act as physical barriers membranes simulate extracellular matrix (ECM) structure assure gas exchange, cell proliferation, and nutrient supply	the materials and solvents used in the production process may be harmful	[48,185]
Fibers	non-adherent high porosity and absorption capacity mimic the skin’s extracellular matrix	unsuitable for third-degree, eschar, and dry wounds if the wound is highly exudative, need a secondary dressing	[205,206]

**Table 5:** Antimicrobial effect of chitosan derivatives demonstrated using the agar diffusion method.

Chitosan-Based Wound Dressing	Degree of Deacetylation %	Pathogenic Microorganism	Inhibition Zone Diameter	Reference
Chitosan/chitin/glucan nonwoven mats	n.a.	*E. coli* (−)	12 mm	[219]
		*K. pneumoniae* (−)	8 mm	
		*S. aureus* (+)	13 mm	
		*Bacillus. subtilis* (+)	11 mm	
Alginate/chitosan-based bilayer composite membrane loaded with ciprofloxacin hydrochloride	n.a.	*E. coli* (−)	pronounced inhibitory effect	[220]
		*P. aeruginosa* (−)		
		*S. aureus* (+)		
Alginate–chitosan hydrogel loaded with tetracycline hydrochloride	n.a.	*E. coli* (−)	10.5 mm	[221]
		*S. aureus* (+)	10 mm	
Copper-incorporated microporous chitosan–polyethylene glycol hydrogels loaded with naproxen	>75%	*E. coli* (−)	pronounced inhibitory effect	[222]
		*S. aureus* (+)		
Chitosan films with *Hypericum perforatum* oil (1% *w/v* chitosan film incorporated with 1.5% *v/v* *H. perforatum* oil)	85%	*E. coli* (−) (20 mm inhibition zone for 0% *H. perforatum* oil films)	20.9 mm	[223]
		*S. aureus* (+) (12.4 mm inhibition zone for 0% *H. perforatum* oil films)	19.7 mm	
Chitosan–AgNO3 hydrogels (CTS-Ag+/NH3)	90%	*E. coli* (−) *ATCC 25922*	obvious antibacterial effect	[68]
		*E. coli* (−) *ATCC 35218*		
		*S. aureus* (+) *ATCC 25923*		
		*S. aureus ssp. aureus* (+) *ATCC29213*		
		*P. aeruginosa* (−) *ATCC 27853*		
		*Enterococcus faecalis ATCC 29212*		
Chitosan–glycerol membrane loaded with Tetracycline Hydrochloride	93%	*E. coli* (−)	7.04 mm	[181]
		*S. aureus* (+)	10.56 mm	
Chitosan–glycerol membrane loaded with silver sulfadiazine		*E. coli* (−)	5.32 mm	
		*S. aureus* (+)	3.52 mm	
Chitosan–Ag nanoparticle bilayer sponge	93.70%	*S. aureus* (+)	3 mm	[224]
		*E. coli* (−)	2 mm	
		*P. aeruginosa* (−)	4 mm	
Quaternized chitosan/polyvinyl alcohol/sodium carboxymethylcellulose blend film		*E. coli* (−)	10.35 ± 0.12 mm	[225]
		*S. aureus* (+)	10.55 ± 0.20 mm	
Chitosan/polyethylene glycol fumarate/thymol hydrogel	75–85%	*E. coli* (−) (1.8% *v/v* Thymol)	6.4 ± 0.9 mm	[226]
		*S. aureus* (+) (1.8% *v/v* Thymol)	10.5 ± 1.8 mm	
Bacterial cellulose–chitosan membranes	90%	*E. coli* (−)	no inhibition zone	[227]
		*S. aureus* (+)	no inhibition zone	

n.a.—data not available.

**Table 6:** Chitosan-based wound dressings on the market.

Product	Dressing Type	Material	Producer
Axiostat^®^	Sponge	100% chitosan	Axiobio
Chitoderm^®^ plus	Superabsorber	Strong superabsorber coated with chitosan	Trusetal
ChitoSAM™ 100	Non-woven chitosan dressing spun directly from chitosan	100% chitosan	Sam Medical
Celox™	Gauze (Celox Rapid, Celox Gauze) Granules (Celox A, Celox Granules)	Chito-R™ activated chitosan granules	MedTrade
ChitoClear^®^	Gel or liquid spray	ChitoClear^®^ positively charged chitosan (the purest chitosan possible)	Primex
Opticell^®^	Gelling fiber	Primarily composed of chitosan (Cytoform chitosan-based gelling technology)	Medline
ChitoFlex^®^ PRO	Hemostatic dressing active on both sides	Chitosan-based dressings	Tricol Biomedical
ChitoGauze^®^ PRO	Chitosan-coated gauze		
ChitoDot^®^	Double-sided hemostatic dressing		
HemCon^®^ Bandage PRO	Hemostatic bandage		
HemCon Patch^®^ PRO	Non-invasive hemostatic patch		
HemCon^®^ Strip PRO	Hemostatic bandage		
KytoCel	Gelling fiber	Chitosan fibers	Aspen Medical
ExcelArrest^®^ XT	Hemostatic patch	MC (modified chitosan)	Hemostasis
PosiSep^®^	Hemostatic sponge	NOCC (N-O-carboxymethyl chitosan)	
HemoPore^®^	Hemostatic bioresorbable nasal dressing	Chitosan lactate	Stryker
XSTAT	Hemostatic device containing superabsorbent sponges of chitosan	Wood pulp sponges coated with chitosan	RevMedX
Alchite (University of Bolton patent)	Composite fiber	Alginate and chitosan	University of Bolton patent
LQD	spray	CHITOSAN-FH02™ a higher positive charge and the highest degree of de-acetylation of any chitosan product	Medoderm GmbH Brancaster Pharma
ChitoHeal	Gel	*N*-acetyl-d-Glucosamine (chitosan)	ChitoTech
ChitoClot Bandage	non-woven dressing	100% chitosan-based, non-woven with adhesive back sheet	BenQ Materials BioMedical
ChitoClot Pad	sponge	100% medical-grade chitosan	
ChitoClot Gauze	gauze		
ChitoRhino	spray	Distilled water, chitosan, xylitol, natural sea salt, grapefruit seed extract, citric acid	Ideoto LLC
	gel	Distilled water, all-natural sea salt, chitosan, xylitol, methylcellulose, aloe vera, grapefruit seed extract, citric acid	

## References

[1] Kingston W. (2008). Irish contributions to the origins of antibiotics. Ir. J. Med. Sci..

[2] Wainwright M. (2008). Some highlights in the history of fungi in medicine—A personal journey. Fungal. Biol. Rev..

[3] Fleming A. (1929). On the antibacterial action of cultures of a penicillium, with special reference to their use in the isolation of B. influenzæ. Br. J. Exp. Pathol..

[4] Durand G.A., Raoult D., Dubourg G. (2019). Antibiotic discovery: History, methods and perspectives. Int. J. Antimicrob. Agents.

[5] Mantravadi P.K., Kalesh K.A., Dobson R.C.J., Hudson A.O., Parthasarathy A. (2019). The Quest for Novel Antimicrobial Compounds: Emerging Trends in Research, Development, and Technologies. Antibiotics (Basel, Switzerland).

[6] Chandra H., Bishnoi P., Yadav A., Patni B., Mishra A.P., Nautiyal A.R. (2017). Antimicrobial Resistance and the Alternative Resources with Special Emphasis on Plant-Based Antimicrobials-A Review. Plants (Basel).

[7] Filius P.M., Gyssens I.C. (2002). Impact of increasing antimicrobial resistance on wound management. Am. J. Clin. Dermatol..

[8] Malone M., Bjarnsholt T., McBain A.J., James G.A., Stoodley P., Leaper D., Tachi M., Schultz G., Swanson T., Wolcott R.D. (2017). The prevalence of biofilms in chronic wounds: A systematic review and meta-analysis of published data. J. Wound Care.

[9] Aleanizy F.S., Alqahtani F.Y., Shazly G., Alfaraj R., Alsarra I., Alshamsan A., Gareeb Abdulhady H. (2018). Measurement and evaluation of the effects of pH gradients on the antimicrobial and antivirulence activities of chitosan nanoparticles in Pseudomonas aeruginosa. Saudi Pharm J..

[10] Omar A., Wright J.B., Schultz G., Burrell R., Nadworny P. (2017). Microbial Biofilms and Chronic Wounds. Microorganisms.

[11] Kadam S., Shai S., Shahane A., Kaushik K.S. (2019). Recent Advances in Non-Conventional Antimicrobial Approaches for Chronic Wound Biofilms: Have We Found the ‘Chink in the Armor’?. Biomedicines.

[12] Hall C.W., Mah T.F. (2017). Molecular mechanisms of biofilm-based antibiotic resistance and tolerance in pathogenic bacteria. FEMS Microbiol. Rev..

[13] Singh S., Singh S.K., Chowdhury I., Singh R. (2017). Understanding the Mechanism of Bacterial Biofilms Resistance to Antimicrobial Agents. Open Microbiol. J..

[14] Miyaue S., Suzuki E., Komiyama Y., Kondo Y., Morikawa M., Maeda S. (2018). Bacterial Memory of Persisters: Bacterial Persister Cells Can Retain Their Phenotype for Days or Weeks After Withdrawal From Colony-Biofilm Culture. Front. Microbiol.

[15] Thurlow L.R., Hanke M.L., Fritz T., Angle A., Aldrich A., Williams S.H., Engebretsen I.L., Bayles K.W., Horswill A.R., Kielian T. (2011). Staphylococcus aureus biofilms prevent macrophage phagocytosis and attenuate inflammation in vivo. J. Immunol..

[16] ECDC European Centre for Disease Prevention and Control. https://www.ecdc.europa.eu/en/news-events/33000-people-die-every-year-due-infections-antibiotic-resistant-bacteria.

[17] Kamaruzzaman N.F., Tan L.P., Hamdan R.H., Choong S.S., Wong W.K., Gibson A.J., Chivu A., Pina M.d.F. (2019). Antimicrobial Polymers: The Potential Replacement of Existing Antibiotics?. Int. J. Mol. Sci..

[18] Huang K.S., Yang C.H., Huang S.-L., Chen C.Y., Lu Y.Y., Lin Y.S. (2016). Recent Advances in Antimicrobial Polymers: A Mini-Review. Int. J. Mol. Sci..

[19] Hosseinnejad M., Jafari S.M. (2016). Evaluation of different factors affecting antimicrobial properties of chitosan. Int. J. Biol. Macromol..

[20] Abdel-Rahman R.M., Hrdina R., Abdel-Mohsen A.M., Fouda M.M.G., Soliman A.Y., Mohamed F.K., Mohsin K., Pinto T.D. (2015). Chitin and chitosan from Brazilian Atlantic Coast: Isolation, characterization and antibacterial activity. Int. J. Biol. Macromol..

[21] Yeul V.S., Rayalu S.S. (2013). Unprecedented chitin and chitosan: A chemical overview. J. Polym. Environ..

[22] Islam S., Bhuiyan M.A.R., Islam M.N. (2017). Chitin and chitosan: Structure, properties and applications in biomedical engineering. J. Polym. Environ..

[23] Kaur S., Dhillon G.S. (2015). Recent trends in biological extraction of chitin from marine shell wastes: A review. Crit. Rev. Biotechnol..

[24] Philibert T., Lee B.H., Fabien N. (2017). Current status and new perspectives on chitin and chitosan as functional biopolymers. Appl. Biochem. Biotechnol..

[25] Fernando L.A.T., Poblete M.R.S., Ongkiko A.G.M., Diaz L.J.L. (2016). Chitin extraction and synthesis of chitin-based polymer films from philippine blue swimming crab (*Portunus pelagicus*) shells. Procedia Chem..

[26] Lopes C., Antelo L.T., Franco-Uría A., Alonso A.A., Pérez-Martín R. (2018). Chitin production from crustacean biomass: Sustainability assessment of chemical and enzymatic processes. J. Clean. Prod..

[27] Kuddus M., Ahmad I.Z. (2013). Isolation of novel chitinolytic bacteria and production optimization of extracellular chitinase. J. Genet. Eng. Biotechnol..

[28] Hamed I., Özogul F., Regenstein J.M. (2016). Industrial applications of crustacean by-products (chitin, chitosan, and chitooligosaccharides): A review. Trends Food Sci. Technol..

[29] Xu Y., Bajaj M., Schneider R., Grage S., Ulrich A., Winter J., Gallert C. (2013). Transformation of the matrix structure of shrimp shells during bacterial deproteination and demineralization. Microb. Cell Fact..

[30] FAO The State of World Fisheries and Aquaculture 2018—Meeting the Sustainable Development Goals, Rome. http://www.fao.org/3/I9540EN/i9540en.pdf.

[31] Gao X., Chen X., Zhang J., Guo W., Jin F., Yan N. (2016). Transformation of chitin and waste shrimp shells into acetic acid and pyrrole. ACS Sustain. Chem. Eng..

[32] Mao X., Guo N., Sun J., Xue C. (2017). Comprehensive utilization of shrimp waste based on biotechnological methods: A review. J. Clean. Prod..

[33] Vázquez J.A., Ramos P., Mirón J., Valcarcel J., Sotelo C.G., Pérez-Martín R.I. (2017). Production of chitin from *Penaeus vannamei* by-products to pilot plant scale using a combination of enzymatic and chemical processes and subsequent optimization of the chemical production of chitosan by response surface methodology. Mar. Drugs.

[34] Younes I., Sellimi S., Rinaudo M., Jellouli K., Nasri M. (2014). Influence of acetylation degree and molecular weight of homogeneous chitosans on antibacterial and antifungal activities. Int. J. Food Microbiol..

[35] Lizardi-Mendoza J., Argüelles Monal W.M., Goycoolea Valencia F.M., Bautista-Baños S., Romanazzi G., Jiménez-Aparicio A. (2016). Chapter 1—Chemical Characteristics and Functional Properties of Chitosan. Chitosan in the Preservation of Agricultural Commodities.

[36] Lozano-Navarro J.I., Diaz-Zavala N.P., Velasco-Santos C., Martinez-Hernandez A.L., Tijerina-Ramos B.I., Garcia-Hernandez M., Rivera-Armenta J.L., Paramo-Garcia U., Reyes-de la Torre A.I. (2017). Antimicrobial, optical and mechanical properties of chitosan-starch films with natural extracts. Int. J. Mol. Sci..

[37] Kean T., Thanou M. (2010). Biodegradation, biodistribution and toxicity of chitosan. Adv. Drug Deliver Rev..

[38] Landriscina A., Rosen J., Friedman A.J. (2015). Biodegradable chitosan nanoparticles in drug delivery for infectious disease. Nanomedicine (Lond.).

[39] Pang Y., Qin A., Lin X., Yang L., Wang Q., Wang Z., Shan Z., Li S., Wang J., Fan S. (2017). Biodegradable and biocompatible high elastic chitosan scaffold is cell-friendly both in vitro and in vivo. Oncotarget.

[40] Matica A., Menghiu G., Ostafe V. (2017). Biodegradability of chitosan based products. New Front. Chem..

[41] Lu Z., Gao J., He Q., Wu J., Liang D., Yang H., Chen R. (2017). Enhanced antibacterial and wound healing activities of microporous chitosan-Ag/ZnO composite dressing. Carbohydr. Polym..

[42] Sayari N., Sila A., Abdelmalek B.E., Abdallah R.B., Ellouz-Chaabouni S., Bougatef A., Balti R. (2016). Chitin and chitosan from the Norway lobster by-products: Antimicrobial and anti-proliferative activities. Int. J. Biol. Macromol..

[43] Cheung R.C.F., Ng T.B., Wong J.H., Chan W.Y. (2015). Chitosan: An update on potential biomedical and pharmaceutical applications. Mar. Drugs.

[44] Huo M., Zhang Y., Zhou J., Zou A., Yu D., Wu Y., Li J., Li H. (2010). Synthesis and characterization of low-toxic amphiphilic chitosan derivatives and their application as micelle carrier for antitumor drug. Int. J. Pharm..

[45] Matica A., Menghiu G., Ostafe V. (2017). Toxicity of chitosan based products. New Front. Chem..

[46] Yadav P., Chaudhary S., Saxena R.K., Talwar S., Yadav S. (2017). Evaluation of antimicrobial and antifungal efficacy of chitosan as endodontic irrigant against *Enterococcus faecalis* and *Candida albicans* biofilm formed on tooth substrate. J. Clin. Exp. Dent..

[47] Wu T., Wu C., Fu S., Wang L., Yuan C., Chen S., Hu Y. (2017). Integration of lysozyme into chitosan nanoparticles for improving antibacterial activity. Carbohydr. Polym..

[48] Zahedi P., Rezaeian I., Ranaei-Siadat S.-O., Jafari S.H., Supaphol P. (2009). A review on wound dressing with an emphasis on electrospun nanofibrous polymeric bandages. Polym. Adv. Technol..

[49] Chen W., Li Y., Yang S., Yue L., Jiang Q., Xia W. (2015). Synthesis and antioxidant properties of chitosan and carboxymethyl chitosan-stabilized selenium nanoparticles. Carbohydr. Polym..

[50] Ren W., Cheng W., Wang G., Liu Y. (2017). Developments in antimicrobial polymers. J. Polym. Sci. Pol. Chem..

[51] Dragostin O.M., Samal S.K., Dash M., Lupascu F., Pânzariu A., Tuchilus C., Ghetu N., Danciu M., Dubruel P., Pieptu D. (2016). New antimicrobial chitosan derivatives for wound dressing applications. Carbohydr. Polym..

[52] Matica A., Menghiu G., Ostafe V. (2017). Antifungal properties of chitosans. New Front. Chem..

[53] Laokuldilok T., Potivas T., Kanha N., Surawang S., Seesuriyachan P., Wangtueai S., Phimolsiripol Y., Regenstein J.M. (2017). Physicochemical, antioxidant, and antimicrobial properties of chitooligosaccharides produced using three different enzyme treatments. Food Biosci.

[54] Hafsa J., Smach M.A., Charfeddine B., Limem K., Majdoub H., Rouatbi S. (2016). Antioxidant and antimicrobial proprieties of chitin and chitosan extracted from *Parapenaeus longirostris* shrimp shell waste. Ann. Pharm. Fr..

[55] López-Mata M.A., Ruiz-Cruz S., de Jesús Ornelas-Paz J., Del Toro-Sánchez C.L., Márquez-Ríos E., Silva-Beltrán N.P., Cira-Chávez L.A., Burruel-Ibarra S.E. (2018). Mechanical barrier and antioxidant properties of chitosan films incorporating cinnamaldehyde. J. Polym. Environ..

[56] Sousa F., Guebitz G.M., Kokol V. (2009). Antimicrobial and antioxidant properties of chitosan enzymatically functionalized with flavonoids. Process. Biochem..

[57] Wimardhani Y.S., Suniarti D.F., Freisleben H.J., Wanandi S.I., Siregar N.C., Ikeda M.A. (2014). Chitosan exerts anticancer activity through induction of apoptosis and cell cycle arrest in oral cancer cells. J. Oral Sci..

[58] Quagliariello V., Masarone M., Armenia E., Giudice A., Barbarisi M., Caraglia M., Barbarisi A., Persico M. (2018). Chitosan-coated liposomes loaded with butyric acid demonstrate anticancer and anti-inflammatory activity in human hepatoma HepG2 cells. Oncol. Rep..

[59] Abruzzo A., Zuccheri G., Belluti F., Provenzano S., Verardi L., Bigucci F., Cerchiara T., Luppi B., Calonghi N. (2016). Chitosan nanoparticles for lipophilic anticancer drug delivery: Development, characterization and in vitro studies on HT29 cancer cells. Colloids Surf. B.

[60] Gibot L., Chabaud S., Bouhout S., Bolduc S., Auger F.A., Moulin V.J. (2015). Anticancer properties of chitosan on human melanoma are cell line dependent. Int. J. Biol. Macromol..

[61] Sadreddini S., Safaralizadeh R., Baradaran B., Aghebati-Maleki L., Hosseinpour-Feizi M.A., Shanehbandi D., Jadidi-Niaragh F., Sadreddini S., Kafil H.S., Younesi V. (2017). Chitosan nanoparticles as a dual drug/siRNA delivery system for treatment of colorectal cancer. Immunol. Lett..

[62] Yadav P., Bandyopadhyay A., Chakraborty A., Sarkar K. (2018). Enhancement of anticancer activity and drug delivery of chitosan-curcumin nanoparticle via molecular docking and simulation analysis. Carbohydr. Polym..

[63] Bano I., Arshad M., Yasin T., Ghauri M.A., Younus M. (2017). Chitosan: A potential biopolymer for wound management. Int. J. Bio.l Macromol..

[64] Rocha M.A.M., Coimbra M.A., Nunes C. (2017). Applications of chitosan and their derivatives in beverages: A critical review. Curr. Opin. Food Sci..

[65] Ribeiro J.C.V., Vieira R.S., Melo I.M., Araujo V.M.A., Lima V. (2017). Versatility of chitosan-based biomaterials and their use as scaffolds for tissue regeneration. Sci. World J..

[66] Mincea M., Patrulea V., Negrulescu A., Szabo R., Ostafe V. (2013). Adsorption of Three Commercial Dyes onto Chitosan Beads Using Spectrophotometric Determination and a Multivariate Calibration Method. JWARP.

[67] Kamel N.A., Abd El-messieh S.L., Saleh N.M. (2017). Chitosan/banana peel powder nanocomposites for wound dressing application: Preparation and characterization. Mater. Sci. Eng. C.

[68] Li P., Zhao J., Chen Y., Cheng B., Yu Z., Zhao Y., Yan X., Tong Z., Jin S. (2017). Preparation and characterization of chitosan physical hydrogels with enhanced mechanical and antibacterial properties. Carbohydr. Polym..

[69] Muzzarelli R., Tarsi R., Filippini O., Giovanetti E., Biagini G., Varaldo P.E. (1990). Antimicrobial properties of N-carboxybutyl chitosan. Antimicrob Agents Chemother..

[70] Leuba J.L., Stossel P., Muzzarelli R., Jeuniaux C., Gooday G.W. (1986). Chitosan and other polyamines: Antifungal activity and interaction with biological membranes. Chitin in Nature and Technology.

[71] Rabea E.I., Badawy M.E.T., Stevens C.V., Smagghe G., Steurbaut W. (2003). Chitosan as antimicrobial agent:  Applications and mode of action. Biomacromolecules.

[72] Goy R.C., Britto D.d., Assis O.B. (2009). A review of the antimicrobial activity of chitosan. Polímeros.

[73] Severino R., Ferrari G., Vu K.D., Donsì F., Salmieri S., Lacroix M. (2015). Antimicrobial effects of modified chitosan based coating containing nanoemulsion of essential oils, modified atmosphere packaging and gamma irradiation against *Escherichia coli* O157:H7 and *Salmonella typhimurium* on green beans. Food Control.

[74] Tamara F.R., Lin C., Mi F.L., Ho Y.C. (2018). Antibacterial Effects of Chitosan/Cationic Peptide Nanoparticles. Nanomaterials Basel.

[75] Beck B., Yildirim Aksoy M., Shoemaker C., Fuller A., Peatman E. (2019). Antimicrobial activity of the biopolymer chitosan against Streptococcus iniae. J. Fish. Dis..

[76] Tao Y., Qian L.H., Xie J. (2011). Effect of chitosan on membrane permeability and cell morphology of *Pseudomonas aeruginosa* and *Staphyloccocus aureus*. Carbohydr. Polym..

[77] Helander I.M., Nurmiaho-Lassila E.L., Ahvenainen R., Rhoades J., Roller S. (2001). Chitosan disrupts the barrier properties of the outer membrane of Gram-negative bacteria. Int. J. Food Microbiol..

[78] Xing K., Zhu X., Peng X., Qin S. (2015). Chitosan antimicrobial and eliciting properties for pest control in agriculture: A review. Agron. Sustain. Dev..

[79] Raafat D., von Bargen K., Haas A., Sahl H.-G. (2008). Insights into the mode of action of chitosan as an antibacterial compound. Appl. Environ. Microbiol..

[80] Pasquina L.W., Santa Maria J.P., Walker S. (2013). Teichoic acid biosynthesis as an antibiotic target. Curr. Opin. Microbiol..

[81] Lee D.S., Je J.Y. (2013). Gallic acid-grafted-chitosan inhibits foodborne pathogens by a membrane damage mechanism. J. Agric. Food Chem..

[82] Xing K., Chen X.G., Kong M., Liu C.S., Cha D.S., Park H.J. (2009). Effect of oleoyl-chitosan nanoparticles as a novel antibacterial dispersion system on viability, membrane permeability and cell morphology of *Escherichia coli* and *Staphylococcus aureus*. Carbohydr. Polym..

[83] Xing K., Chen X.G., Liu C.S., Cha D.S., Park H.J. (2009). Oleoyl-chitosan nanoparticles inhibits *Escherichia coli* and *Staphylococcus aureus* by damaging the cell membrane and putative binding to extracellular or intracellular targets. J. Agric. Food Chem..

[84] Park S.C., Nam J.P., Kim J.H., Kim Y.M., Nah J.W., Jang M.K. (2015). Antimicrobial action of water-soluble β-chitosan against clinical multi-drug resistant bacteria. Int. J. Mol. Sci..

[85] Wang Q.Z., Chen X.G., Liu N., Wang S.X., Liu C.S., Meng X.H., Liu C.G. (2006). Protonation constants of chitosan with different molecular weight and degree of deacetylation. Carbohydr. Polym..

[86] Szymańska E., Winnicka K. (2015). Stability of chitosan-a challenge for pharmaceutical and biomedical applications. Mar. Drugs.

[87] Liu H., Du Y., Wang X., Sun L. (2004). Chitosan kills bacteria through cell membrane damage. Int. J. Food Microbiol..

[88] Fu X., Shen Y., Jiang X., Huang D., Yan Y. (2011). Chitosan derivatives with dual-antibacterial functional groups for antimicrobial finishing of cotton fabrics. Carbohydr. Polym..

[89] Li X.F., Feng X.G., Yang S., Fu G.G., Wang T.P., Su Z.X. (2010). Chitosan kills *Escherichia coli* through damage to be of cell membrane mechanism. Carbohydr. Polym..

[90] Schoukens G., Rajendran S. (2009). 5—Bioactive dressings to promote wound healing. Advanced Textiles for Wound Care.

[91] Verlee A., Mincke S., Stevens C.V. (2017). Recent developments in antibacterial and antifungal chitosan and its derivatives. Carbohydr. Polym..

[92] Palma-Guerrero J., Lopez-Jimenez J.A., Perez-Berna A.J., Huang I.C., Jansson H.B., Salinas J., Villalain J., Read N.D., Lopez-Llorca L.V. (2010). Membrane fluidity determines sensitivity of filamentous fungi to chitosan. Mol. Microbiol..

[93] Yuan G., Lv H., Tang W., Zhang X., Sun H. (2016). Effect of chitosan coating combined with pomegranate peel extract on the quality of Pacific white shrimp during iced storage. Food Control..

[94] Archana D., Singh B.K., Dutta J., Dutta P.K. (2015). Chitosan-PVP-nano silver oxide wound dressing: In vitro and in vivo evaluation. Int. J. Biol. Macromol..

[95] Galván Márquez I., Akuaku J., Cruz I., Cheetham J., Golshani A., Smith M.L. (2013). Disruption of protein synthesis as antifungal mode of action by chitosan. Int. J. Food Microbiol..

[96] Mansilla A.Y., Albertengo L., Rodríguez M.S., Debbaudt A., Zúñiga A., Casalongué C.A. (2013). Evidence on antimicrobial properties and mode of action of a chitosan obtained from crustacean exoskeletons on *Pseudomonas syringae* pv. *tomato* DC3000. Appl. Microbiol. Biot..

[97] Park S.C., Nah J.W., Park Y. (2011). pH-dependent mode of antibacterial actions of low molecular weight water-soluble chitosan (LMWSC) against various pathogens. Macromol. Res..

[98] Chien R.C., Yen M.T., Mau J.L. (2016). Antimicrobial and antitumor activities of chitosan from shiitake stipes, compared to commercial chitosan from crab shells. Carbohydr. Polym..

[99] Matica A., Menghiu G., Ostafe V. (2017). Antibacterial properties of chitin and chitosans. New Front. Chem..

[100] Kong M., Chen X.G., Xing K., Park H.J. (2010). Antimicrobial properties of chitosan and mode of action: A state of the art review. Int. J. Food Microbiol..

[101] Clifton L.A., Skoda M.W., Le Brun A.P., Ciesielski F., Kuzmenko I., Holt S.A., Lakey J.H. (2015). Effect of divalent cation removal on the structure of Gram-negative bacterial outer membrane models. Langmuir.

[102] Kakaei S., Shahbazi Y. (2016). Effect of chitosan-gelatin film incorporated with ethanolic red grape seed extract and *Ziziphora clinopodioides* essential oil on survival of *Listeria monocytogenes* and chemical, microbial and sensory properties of minced trout fillet. Lwt-Food Sci. Technol..

[103] Nowzari F., Shábanpour B., Ojagh S.M. (2013). Comparison of chitosan–gelatin composite and bilayer coating and film effect on the quality of refrigerated rainbow trout. Food Chem..

[104] Mohan C.O., Ravishankar C.N., Lalitha K.V., Srinivasa Gopal T.K. (2012). Effect of chitosan edible coating on the quality of double filleted Indian oil sardine (*Sardinella longiceps)* during chilled storage. Food Hydrocoll..

[105] El-tahlawy K.F., El-bendary M.A., Elhendawy A.G., Hudson S.M. (2005). The antimicrobial activity of cotton fabrics treated with different crosslinking agents and chitosan. Carbohydr. Polym..

[106] Devlieghere F., Vermeulen A., Debevere J. (2004). Chitosan: Antimicrobial activity, interactions with food components and applicability as a coating on fruit and vegetables. Food Microbiol..

[107] Tayel A.A., Moussa S., Opwis K., Knittel D., Schollmeyer E., Nickisch-Hartfiel A. (2010). Inhibition of microbial pathogens by fungal chitosan. Int. J. Biol. Macromol..

[108] Champer J., Patel J., Fernando N., Salehi E., Wong V., Kim J. (2013). Chitosan against cutaneous pathogens. AMB Express..

[109] Oliveira Junior E., Melo I., Franco T. (2012). Changes in hyphal morphology due to chitosan treatment in some fungal species. Braz. Arch. Biol. Techn..

[110] Davidova V.N., Naberezhnykh G.A., Yermak I.M., Gorbach V.I., Solov’eva T.F. (2006). Determination of binding constants of lipopolysaccharides of different structure with chitosan. Biochemistry (Moscow.).

[111] Tacconelli E., Carrara E., Savoldi A., Harbarth S., Mendelson M., Monnet D.L., Pulcini C., Kahlmeter G., Kluytmans J., Carmeli Y. (2018). Discovery, research, and development of new antibiotics: The WHO priority list of antibiotic-resistant bacteria and tuberculosis. Lancet. Infect. Dis..

[112] Caetano G., Frade M., Andrade T., Leite M., Zorzi Bueno C., Moraes Â., Ribeiro-Paes J.T. (2014). Chitosan-alginate membranes accelerate wound healing. J. Biomed. Mater. Res. B.

[113] Han G., Ceilley R. (2017). Chronic wound healing: A review of current management and treatments. Adv. Ther..

[114] Nikaido H. (2003). Molecular basis of bacterial outer membrane permeability revisited. Microbiol. Mol. Biol. R.

[115] Seyfarth F., Schliemann S., Elsner P., Hipler U.C. (2008). Antifungal effect of high- and low-molecular-weight chitosan hydrochloride, carboxymethyl chitosan, chitosan oligosaccharide and *N*-acetyl-d-glucosamine against *Candida albicans*, *Candida krusei* and *Candida glabrata*. Int. J. Pharmaceut..

[116] Lopez-Moya F., Suarez-Fernandez M., Lopez-Llorca L.V. (2019). Molecular mechanisms of chitosan interactions with fungi and plants. Int. J. Mol. Sci..

[117] Nwe N., Furuike T., Tamura H., Jayakumar R., Prabaharan M., Muzzarelli R.A.A. (2011). Production, properties and applications of fungal cell wall polysaccharides: Chitosan and glucan. Chitosan for Biomaterials II.

[118] Masuoka J. (2004). Surface glycans of *Candida albicans* and other pathogenic fungi: Physiological roles, clinical uses, and experimental challenges. Clin. Microbiol. Rev..

[119] Tronchin G., Pihet M., Lopes-Bezerra L.M., Bouchara J.P. (2008). Adherence mechanisms in human pathogenic fungi. Med. Mycol..

[120] Soares R.M.A., de A. Soares R.M., Soares R.M., Alviano D.S., Angluster J., Alviano C.S., Travassos L.R. (2000). Identification of sialic acids on the cell surface of *Candida albicans*. Biochim. Biophys. Acta.

[121] Alviano C.S., Travassos L.R., Schauer R. (1999). Sialic acids in fungi. Glycoconj. J..

[122] Yien Ing L., Zin N., Sarwar A., Katas H. (2012). Antifungal activity of chitosan nanoparticles and correlation with their physical properties. Int. J. Biomater..

[123] Peña A., Sánchez N.S., Calahorra M. (2013). Effects of chitosan on *Candida albicans*: Conditions for its antifungal activity. Biomed. Res. Int..

[124] Sobhani Z., Samani S., Montaseri H., Khezri E. (2017). Nanoparticles of chitosan loaded ciprofloxacin: Fabrication and antimicrobial activity. Adv. Pharm. Bull..

[125] Liu X., Xia W., Jiang Q., Yu P., Yue L. (2018). Chitosan oligosaccharide-*N*-chlorokojic acid mannich base polymer as a potential antibacterial material. Carbohydr. Polym..

[126] Burkatovskaya M., Castano A.P., Demidova-Rice T.N., Tegos G.P., Hamblin M.R. (2008). Effect of chitosan acetate bandage on wound healing in infected and noninfected wounds in mice. Wound Repair. Regen..

[127] Dash M., Chiellini F., Ottenbrite R.M., Chiellini E. (2011). Chitosan—A versatile semi-synthetic polymer in biomedical applications. Prog. Polym. Sci..

[128] Liu H., Wang C., Li C., Qin Y., Wang Z., Yang F., Li Z., Wang J. (2018). A functional chitosan-based hydrogel as a wound dressing and drug delivery system in the treatment of wound healing. RSC Adv..

[129] Shi C., Zhu Y., Ran X., Wang M., Su Y., Cheng T. (2006). Therapeutic potential of chitosan and its derivatives in regenerative medicine. J. Surg. Res..

[130] Sharma D., Rajput J., Kaith B.S., Kaur M., Sharma S. (2010). Synthesis of ZnO nanoparticles and study of their antibacterial and antifungal properties. Thin Solid Films.

[131] Diegelmann R.F., Dunn J.D., Lindblad W.J., Cohen I.K. (1996). Analysis of the effects of chitosan on inflammation, angiogenesis, fibroplasia, and collagen deposition in polyvinyl alcohol sponge implants in rat wounds. Wound Repair Regen..

[132] Kosaka T., Kaneko Y., Nakada Y., Matsuura M., Tanaka S. (1996). Effect of chitosan implantation on activation of canine macrophages and polymorphonuclear cells after surgical stress. J. Vet. Med. Sci..

[133] Howling G.I., Dettmar P.W., Goddard P.A., Hampson F.C., Dornish M., Wood E.J. (2001). The effect of chitin and chitosan on the proliferation of human skin fibroblasts and keratinocytes in vitro. Biomaterials.

[134] Mori T., Okumura M., Matsuura M., Ueno K., Tokura S., Okamoto Y., Minami S., Fujinaga T. (1997). Effects of chitin and its derivatives on the proliferation and cytokine production of fibroblasts in vitro. Biomaterials.

[135] Patrulea V., Applegate L.A., Ostafe V., Jordan O., Borchard G. (2015). Optimized synthesis of O-carboxymethyl-*N*,*N*,*N*-trimethyl chitosan. Carbohydr. Polym..

[136] Patrulea V., Laurent-Applegate L.A., Ostafe V., Borchard G., Jordan O. (2019). Polyelectrolyte nanocomplexes based on chitosan derivatives for wound healing application. Eur. J. Pharm. Biopharm..

[137] Chen Z., Han L., Liu C., Du Y., Hu X., Du G., Shan C., Yang K., Wang C., Li M. (2018). A rapid hemostatic sponge based on large, mesoporous silica nanoparticles and N-alkylated chitosan. Nanoscale.

[138] Maksym P., Sikora V. (2015). Chitosan as a Hemostatic Agent: Current State. Eur. J. Med. Ser. B.

[139] Ong S.Y., Wu J., Moochhala S.M., Tan M.H., Lu J. (2008). Development of a chitosan-based wound dressing with improved hemostatic and antimicrobial properties. Biomaterials.

[140] Periayah M.H., Halim A.S., Yaacob N.S., Saad A.Z.M., Hussein A.R., Rashid A.H.A., Ujang Z. (2014). Glycoprotein IIb/IIIa and P2Y12 induction by oligochitosan accelerates platelet aggregation. Biomed. Res. Int..

[141] Allan G.G., Altman L.C., Bensinger R.E., Ghosh D.K., Hirabayashi Y., Neogi A.N., Neogi S., Zikakis J.P. (1984). Biomedical applications of chitin and chitosan. Chitin, Chitosan, and Related Enzymes.

[142] Huang S., Han B., Shao K., Yu M., Liu W. (2014). Analgesis and wound healing effect of chitosan and carboxymethyl chitosan on scalded rats. J. Ocean Univ. China.

[143] Chen F., Li X., Mo X., He C., Wang H., Ikada Y. (2008). Electrospun chitosan-P(LLA-CL) nanofibers for biomimetic extracellular matrix. J. Biomater. Sci. Polym. E.

[144] Karakeçili A.G., Satriano C., Gümüşderelioğlu M., Marletta G. (2008). Enhancement of fibroblastic proliferation on chitosan surfaces by immobilized epidermal growth factor. Acta Biomater..

[145] Bueter C.L., Specht C.A., Levitz S.M. (2013). Innate sensing of chitin and chitosan. PLoS Pathog..

[146] Brodaczewska K., Wolaniuk N., Lewandowska K., Donskow-Łysoniewska K., Doligalska M. (2017). Biodegradable Chitosan Decreases the Immune Response to Trichinella spiralis in Mice. Molecules.

[147] Bueter C.L., Lee C.K., Rathinam V.A., Healy G.J., Taron C.H., Specht C.A., Levitz S.M. (2011). Chitosan but not chitin activates the inflammasome by a mechanism dependent upon phagocytosis. J. Biolog. Chem..

[148] Wu T., Zivanovic S. (2008). Determination of the degree of acetylation (DA) of chitin and chitosan by an improved first derivative UV method. Carbohydr. Polym..

[149] Kumirska J., Weinhold M., Thöming J., Stepnowski P. (2011). Biomedical activity of chitin/chitosan based materials—Influence of physicochemical properties apart from molecular weight and degree of n-acetylation. Polymers.

[150] Nguyen T., Nguyen D.H., Pham D., Phu D., Hien N., Hoang D. (2017). New oligochitosan-nanosilica hybrid materials: Preparation and application on chili plants for resistance to anthracnose disease and growth enhancement. Polym. J..

[151] Liu X., Howard K.A., Dong M., Andersen M.Ø., Rahbek U.L., Johnsen M.G., Hansen O.C., Besenbacher F., Kjems J. (2007). The influence of polymeric properties on chitosan/siRNA nanoparticle formulation and gene silencing. Biomaterials.

[152] Lee M., Nah J.W., Kwon Y., Koh J.J., Ko K.S., Kim S.W. (2001). Water-soluble and low molecular weight chitosan-based plasmid DNA delivery. Pharm. Res..

[153] Ribeiro L.N., Alcantara A.C., Darder M., Aranda P., Araujo-Moreira F.M., Ruiz-Hitzky E. (2014). Pectin-coated chitosan-LDH bionanocomposite beads as potential systems for colon-targeted drug delivery. Int. J. Pharm..

[154] Qinna N.A., Karwi Q.G., Al-Jbour N., Al-Remawi M.A., Alhussainy T.M., Al-So’ud K.A., Al Omari M.M.H., Badwan A.A. (2015). Influence of molecular weight and degree of deacetylation of low molecular weight chitosan on the bioactivity of oral insulin preparations. Mar. Drugs.

[155] Sugiyanti D., Darmadji P., Santoso U., Pranoto Y., Anwar C., Anggrahini S. (2018). Biological Activity of Native and Low Molecular Weight Chitosan obtained by Steam Explosion Process. Pak. J. Biol. Sci..

[156] Tan G., Kaya M., Tevlek A., Sargin I., Baran T. (2018). Antitumor activity of chitosan from mayfly with comparison to commercially available low, medium and high molecular weight chitosans. In Vitro Cell Dev. Biol. Anim..

[157] Grobler S. (2018). Cytotoxicity of Low, Medium and High Molecular weight Chitosan’s on Balb/c 3T3 Mouse Fibroblast Cells at a 75-85% De-acetylation Degree. Mater. Sci. Eng. Adv. Res..

[158] O’Callaghan K.A.M., Kerry J.P. (2016). Preparation of low- and medium-molecular weight chitosan nanoparticles and their antimicrobial evaluation against a panel of microorganisms, including cheese-derived cultures. Food Control..

[159] Kulikov S.N., Tikhonov V.E., Bezrodnykh E.A., Lopatin S.A., Varlamov V.P. (2015). Comparative evaluation of antimicrobial activity of oligochitosans against *Klebsiella pneumoniae*. Russ. J. Bioorg. Chem..

[160] Tin S., Sakharkar K.R., Lim C.S., Sakharkar M.K. (2009). Activity of Chitosans in combination with antibiotics in Pseudomonas aeruginosa. Int. J. Biol. Sci..

[161] Batista A., Dantas G.C., Santos J., Amorim R.V. (2011). Antimicrobial effects of native chitosan against opportunistic Gram-negative bacteria. Microbiol. J..

[162] Tayel A.A., Moussa S., El-Tras W.F., Knittel D., Opwis K., Schollmeyer E. (2010). Anticandidal action of fungal chitosan against *Candida albicans*. Int. J. Biol. Macromol..

[163] Aliasghari A., Rabbani Khorasgani M., Vaezifar S., Rahimi F., Younesi H., Khoroushi M. (2016). Evaluation of antibacterial efficiency of chitosan and chitosan nanoparticles on cariogenic streptococci: An in vitro study. Iran. J. Microbiol..

[164] Aisverya S., Venkatesan J., Anil S., Kim S.K., Ahmed S., Sudha P.N. (2017). Antimicrobial Competence of Prepared Chitosan-Based Composites. J. Adv. Mater..

[165] Jayakumar R., Prabaharan M., Nair S.V., Tokura S., Tamura H., Selvamurugan N. (2010). Novel carboxymethyl derivatives of chitin and chitosan materials and their biomedical applications. Prog. Mater. Sci..

[166] Jeong Y.I., Kim D.G., Jang M.K., Nah J.W. (2008). Preparation and spectroscopic characterization of methoxy poly(ethylene glycol)-grafted water-soluble chitosan. Carbohydr. Res..

[167] Singh J., Dutta P. (2011). Antibacterial and physiochemical behavior of prepared chitosan/pyridine-3,5-di-carboxylic acid complex for biomedical applications. J. Macromol. Sci. A.

[168] Erdem B., Kaya T., Tulumoglu S., Görgülü Ö. (2016). Factors ınfluencing antibacterial activity of chitosan against *Aeromonas hydrophila* and *Staphylococcus aureus*. Int. Curr. Pharm. J..

[169] Alburquenque C., Bucarey S.A., Neira-Carrillo A., Urzua B., Hermosilla G., Tapia C.V. (2010). Antifungal activity of low molecular weight chitosan against clinical isolates of *Candida* spp.. Med. Mycol..

[170] Tajdini F., Amini M.A., Nafissi-Varcheh N., Faramarzi M.A. (2010). Production, physiochemical and antimicrobial properties of fungal chitosan from *Rhizomucor miehei* and *Mucor racemosus*. Int. J. Biol. Macromol..

[171] Moussa S., Tayel A., Al-Hassan A., Farouk A. (2013). Tetrazolium/formazan test as an efficient method to determine fungal chitosan antimicrobial activity. J. Mycol..

[172] Kumaresapillai N., Ameer Basha R., Sathish R. (2011). Production and evaluation of chitosan from *Aspergillus niger* MTCC strains. Iran. J. Pharm. Res..

[173] Younes I., Rinaudo M. (2015). Chitin and chitosan preparation from marine sources. Structure, properties and applications. Mar. Drugs.

[174] Lee Y., Kim H.W., Brad Kim Y.H. (2017). New route of chitosan extraction from blue crabs and shrimp shells as flocculants on soybean solutes. Food Sci. Biotechnol..

[175] Goy R.C., Morais S.T.B., Assis O.B.G. (2016). Evaluation of the antimicrobial activity of chitosan and its quaternized derivative on *E. coli* and *S. aureus* growth. Rev. Bras. Farmacogn..

[176] Hipalaswins W.M., Balakumaran M., Jagadeeswari S. (2016). Synthesis, characterization and antibacterial activity of chitosan nanoparticles and its impact on seed germination. J. Acad. Ind. Res..

[177] Fernandes J.C., Tavaria F.K., Fonseca S.C., Ramos O.S., Pintado M.E., Malcata F.X. (2010). In vitro screening for anti-microbial activity of chitosans and chitooligosaccharides, aiming at potential uses in functional textiles. J. Microbiol. Biotechnol..

[178] Chung Y.C., Su Y.P., Chen C.C., Jia G., Wang H.L., Wu J.C., Lin J.G. (2004). Relationship between antibacterial activity of chitosan and surface characteristics of cell wall. Acta Pharmacol. Sin..

[179] Zarayneh S., Sepahi A.A., Jonoobi M., Rasouli H. (2018). Comparative antibacterial effects of cellulose nanofiber, chitosan nanofiber, chitosan/cellulose combination and chitosan alone against bacterial contamination of Iranian banknotes. Int. J. Biol. Macromol..

[180] Biswas D.P., O’Brien-Simpson N.M., Reynolds E.C., O’Connor A.J., Tran P.A. (2018). Comparative study of novel in situ decorated porous chitosan-selenium scaffolds and porous chitosan-silver scaffolds towards antimicrobial wound dressing application. J. Colloid Int. Sci..

[181] Ma Y., Xin L., Tan H., Fan M., Li J., Jia Y., Ling Z., Chen Y., Hu X. (2017). Chitosan membrane dressings toughened by glycerol to load antibacterial drugs for wound healing. Mater. Sci. Eng. C Mater. Biol. Appl..

[182] Kaygusuz H., Torlak E., Akin-Evingur G., Ozen I., von Klitzing R., Erim F.B. (2017). Antimicrobial cerium ion-chitosan crosslinked alginate biopolymer films: A novel and potential wound dressing. Int. J. Biol. Macromol..

[183] Anjum S., Arora A., Alam M.S., Gupta B. (2016). Development of antimicrobial and scar preventive chitosan hydrogel wound dressings. Int. J. Pharm..

[184] Wahid F., Wang H.S., Zhong C., Chu L.Q. (2017). Facile fabrication of moldable antibacterial carboxymethyl chitosan supramolecular hydrogels cross-linked by metal ions complexation. Carbohydr. Polym..

[185] Bhattarai S.R., Bhattarai N., Yi H.K., Hwang P.H., Cha D.I., Kim H.Y. (2004). Novel biodegradable electrospun membrane: Scaffold for tissue engineering. Biomaterials.

[186] Park Y.J., Lee H.K. (2018). The Role of Skin and Orogenital Microbiota in Protective Immunity and Chronic Immune-Mediated Inflammatory Disease. Front. Immunol.

[187] Coates M., Blanchard S., MacLeod A. (2018). Innate antimicrobial immunity in the skin: A protective barrier against bacteria, viruses, and fungi. PLoS Pathog..

[188] Dhivya S., Padma V., Elango S. (2015). Wound dressings—A review. BioMedicine.

[189] Jones V., Grey J.E., Harding K.G. (2006). Wound dressings. BMJ.

[190] Ibrahim N.I., Wong S.K., Mohamed I.N., Mohamed N., Chin K.Y., Ima-Nirwana S., Shuid A.N. (2018). Wound healing properties of selected natural products. Int. J. Environ. Res. Public Health.

[191] Boateng J.S., Matthews K.H., Stevens H.N., Eccleston G.M. (2008). Wound healing dressings and drug delivery systems: A review. J. Pharm. Sci..

[192] Simoes D., Miguel S.P., Ribeiro M.P., Coutinho P., Mendonca A.G., Correia I.J. (2018). Recent advances on antimicrobial wound dressing: A. review. Eur. J. Pharm. Biopharm..

[193] Abdelrahman T., Newton H. (2011). Wound dressings: Principles and practice. Surgery (Oxford).

[194] Al-Gharibi K.A., Sharstha S., Al-Faras M.A. (2018). Cost-Effectiveness of Wound Care: A concept analysis. Sultan. Qaboos. Univ. Med. J..

[195] Cui L., Gao S., Song X., Huang L., Dong H., Liu J., Chen F., Yu S. (2018). Preparation and characterization of chitosan membranes. RSC Adv..

[196] Azad A.K., Sermsintham N., Chandrkrachang S., Stevens W.F. (2004). Chitosan membrane as a wound-healing dressing: Characterization and clinical application. J. Biomed. Mater. Res. B Appl. Biomater..

[197] Miguel S.P., Moreira A.F., Correia I.J. (2019). Chitosan based-asymmetric membranes for wound healing: A review. Int. J. Biol. Macromol..

[198] Dai T., Tanaka M., Huang Y.Y., Hamblin M.R. (2011). Chitosan preparations for wounds and burns: Antimicrobial and wound-healing effects. Expert Rev. Anti. Infect. Ther..

[199] Patel S., Srivastava S., Singh M.R., Singh D. (2018). Preparation and optimization of chitosan-gelatin films for sustained delivery of lupeol for wound healing. Int. J. Biol. Macromol..

[200] Yassue-Cordeiro P.H., Severino P., Souto E.M., Pedroso Yoshida C.M., Ferreira da Silva C., Hasnain M.S., Nayak A.K. (2019). Chapter 24—Natural polysaccharides in wound dressing applications. Natural Polysaccharides in Drug Delivery and Biomedical Applications.

[201] Ramos-e-Silva M., Ribeiro de Castro M.C. (2002). New dressings, including tissue-engineered living skin. Clin. Dermatol..

[202] Jayakumar R., Prabaharan M., Sudheesh Kumar P.T., Nair S.V., Tamura H. (2011). Biomaterials based on chitin and chitosan in wound dressing applications. Biotechnol. Adv..

[203] Ahmed E.M. (2015). Hydrogel: Preparation, characterization, and applications: A review. J. Adv. Res..

[204] Pott F.S., Meier M.J., Stocco J.G.D., Crozeta K., Ribas J.D. (2014). The effectiveness of hydrocolloid dressings versus other dressings in the healing of pressure ulcers in adults and older adults: A systematic review and meta-analysis. Rev. Lat. Am. Enfermagem..

[205] Croisier F., Jérôme C. (2013). Chitosan-based biomaterials for tissue engineering. Eur. Polym. J..

[206] Pilehvar-Soltanahmadi Y., Dadashpour M., Mohajeri A., Fattahi A., Sheervalilou R., Zarghami N. (2018). An overview on application of natural substances incorporated with electrospun nanofibrous scaffolds to development of innovative wound dressings. Mini Rev. Med. Chem..

[207] Kenawy el R., Worley S.D., Broughton R. (2007). The chemistry and applications of antimicrobial polymers: A state-of-the-art review. Biomacromolecules.

[208] Kołodyńska D. (2012). Adsorption characteristics of chitosan modified by chelating agents of a new generation. Chem. Eng. J..

[209] Tabriz A., Alvi M.A.U.R., Niazi M.B.K., Batool M., Bhatti M.F., Khan A.L., Khan A.U., Jamil T., Ahmad N.M. (2019). Quaternized trimethyl functionalized chitosan based antifungal membranes for drinking water treatment. Carbohydr. Polym..

[210] Kumar S., Ye F., Dobretsov S., Dutta J. (2019). Chitosan nanocomposite coatings for food, paints, and water treatment applications. App. Sci..

[211] Li X., Sun J., Che Y., Lv Y., Liu F. (2019). Antibacterial properties of chitosan chloride-graphene oxide composites modified quartz sand filter media in water treatment. Int. J. Biol. Macromol..

[212] Rambabu K., Bharath G., Banat F., Show P.L., Cocoletzi H.H. (2019). Mango leaf extract incorporated chitosan antioxidant film for active food packaging. Int. J. Biol. Macromol..

[213] Barra A., Ferreira N.M., Martins M.A., Lazar O., Pantazi A., Jderu A.A., Neumayer S.M., Rodriguez B.J., Enăchescu M., Ferreira P. (2019). Eco-friendly preparation of electrically conductive chitosan—Reduced graphene oxide flexible bionanocomposites for food packaging and biological applications. Compos. Sci. Technol..

[214] Gatto M., Ochi D., Yoshida C.M.P., da Silva C.F. (2019). Study of chitosan with different degrees of acetylation as cardboard paper coating. Carbohydr. Polym..

[215] Habibie S., Hamzah M., Anggaravidya M., Kalembang E. (2016). The effect of chitosan on physical and mechanical properties of paper. J. Chem. Eng. Mater. Sci..

[216] Dias A.M.A., Rey-Rico A., Oliveira R.A., Marceneiro S., Alvarez-Lorenzo C., Concheiro A., Júnior R.N.C., Braga M.E.M., de Sousa H.C. (2013). Wound dressings loaded with an anti-inflammatory jucá (*Libidibia ferrea*) extract using supercritical carbon dioxide technology. J. Supercrit. Fluid..

[217] Azuma K., Izumi R., Osaki T., Ifuku S., Morimoto M., Saimoto H., Minami S., Okamoto Y. (2015). Chitin, chitosan, and its derivatives for wound healing: Old and new materials. J. Funct. Biomater..

[218] Allan C.R., Hadwiger L.A. (1979). The fungicidal effect of chitosan on fungi of varying cell wall composition. Exp. Mycol..

[219] Abdel-Mohsen A.M., Jancar J., Massoud D., Fohlerova Z., Elhadidy H., Spotz Z., Hebeish A. (2016). Novel chitin/chitosan-glucan wound dressing: Isolation, characterization, antibacterial activity and wound healing properties. Int. J. Pharm..

[220] Han F., Dong Y., Song A., Yin R., Li S. (2014). Alginate/chitosan based bi-layer composite membrane as potential sustained-release wound dressing containing ciprofloxacin hydrochloride. Appl. Surf. Sci..

[221] Chen H., Xing X., Tan H., Jia Y., Zhou T., Chen Y., Ling Z., Hu X. (2017). Covalently antibacterial alginate-chitosan hydrogel dressing integrated gelatin microspheres containing tetracycline hydrochloride for wound healing. Mater. Sci. Eng. C Mater. Biol. Appl..

[222] Mishra S.K., Mary D.S., Kannan S. (2017). Copper incorporated microporous chitosan-polyethylene glycol hydrogels loaded with naproxen for effective drug release and anti-infection wound dressing. Int. J. Biol. Macromol..

[223] Gunes S., Tihminlioglu F. (2017). Hypericum perforatum incorporated chitosan films as potential bioactive wound dressing material. Int. J. Biol. Macromol..

[224] Ding L., Shan X., Zhao X., Zha H., Chen X., Wang J., Cai C., Wang X., Li G., Hao J. (2017). Spongy bilayer dressing composed of chitosan-Ag nanoparticles and chitosan-Bletilla striata polysaccharide for wound healing applications. Carbohydr. Polym..

[225] Hu D., Qiang T., Wang L. (2017). Quaternized chitosan/polyvinyl alcohol/sodium carboxymethylcellulose blend film for potential wound dressing application. Wound Med..

[226] Koosehgol S., Ebrahimian-Hosseinabadi M., Alizadeh M., Zamanian A. (2017). Preparation and characterization of in situ chitosan/polyethylene glycol fumarate/thymol hydrogel as an effective wound dressing. Mater. Sci. Eng. C.

[227] Lin W.C., Lien C.C., Yeh H.J., Yu C.M., Hsu S.H. (2013). Bacterial cellulose and bacterial cellulose–chitosan membranes for wound dressing applications. Carbohydr. Polym..

[228] Qasim S.B., Zafar M.S., Najeeb S., Khurshid Z., Shah A.H., Husain S., Rehman I.U. (2018). Electrospinning of chitosan-based solutions for tissue engineering and regenerative medicine. Int. J. Mol. Sci..

[229] Zhou Z., Yan D., Cheng X., Kong M., Liu Y., Feng C., Chen X. (2016). Biomaterials based on *N*,*N*,*N*-trimethyl chitosan fibers in wound dressing applications. Int. J. Biol. Macromol..

[230] Sood A., Granick M.S., Tomaselli N.L. (2014). Wound Dressings and Comparative Effectiveness Data. Adv. Wound Care.

[231] Hiranpattanakul P., Jongjitpissamai T., Aungwerojanawit S., Tachaboonyakiat W. (2018). Fabrication of a chitin/chitosan hydrocolloid wound dressing and evaluation of its bioactive properties. Res. Chem. Intermed..

[232] Pranantyo D., Xu L.Q. (2018). Chitosan-based peptidopolysaccharides as cationic antimicrobial agents and antibacterial coatings. Biomacromolecules.

[233] Garrido-Maestu A., Ma Z., Paik S.-Y.-R., Chen N., Ko S., Tong Z., Jeong K.C. (2018). Engineering of chitosan-derived nanoparticles to enhance antimicrobial activity against foodborne pathogen *Escherichia coli* O157:H7. Carbohydr. Polym..

[234] Anisha B.S., Biswas R., Chennazhi K.P., Jayakumar R. (2013). Chitosan-hyaluronic acid/nano silver composite sponges for drug resistant bacteria infected diabetic wounds. Int. J. Biol. Macromol..

[235] Shao W., Wu J., Wang S., Huang M., Liu X., Zhang R. (2017). Construction of silver sulfadiazine loaded chitosan composite sponges as potential wound dressings. Carbohydr. Polym..

[236] Pei Z., Sun Q., Sun X., Wang Y., Zhao P. (2015). Preparation and characterization of silver nanoparticles on silk fibroin/carboxymethylchitosan composite sponge as anti-bacterial wound dressing. Bio. Med. Mater. Eng..

[237] Mi F.L., Shyu S.S., Wu Y.B., Lee S.T., Shyong J.Y., Huang R.N. (2001). Fabrication and characterization of a sponge-like asymmetric chitosan membrane as a wound dressing. Biomaterials.

[238] Yanagibayashi S., Kishimoto S., Ishihara M., Murakami K., Aoki H., Takikawa M., Fujita M., Sekido M., Kiyosawa T. (2012). Novel hydrocolloid-sheet as wound dressing to stimulate healing-impaired wound healing in diabetic db/db mice. Bio-Med. Mater. Eng..

[239] Trusetal Chitoderm Plus. http://www.trusetal.cl/#.

[240] Celox Celox Medical. https://www.celoxmedical.com/eur/about-celox/.

[241] Medline Opticell. https://www.medline.com/product/Opticell-Gelling-Fiber-Wound-Dressings/Z05-PF50473.

[242] LQD LQD Spray. https://lqdspray.com/.

[243] Wedmore I., McManus J.G., Pusateri A.E., Holcomb J.B. (2006). A special report on the chitosan-based hemostatic dressing: Experience in current combat operations. J. Trauma.

[244] Baldrick P. (2010). The safety of chitosan as a pharmaceutical excipient. Regul. Toxicol. Pharmacol..

[245] Nilsen-Nygaard J., Strand S., Varum K., Draget K., Nordgard C. (2015). Chitosan: Gels and Interfacial Properties. Polymers.

[246] Casettari L., Illum L. (2014). Chitosan in nasal delivery systems for therapeutic drugs. J. Control. Release Off. J. Controll. Release Soc..

[247] Shive M.S., Stanish W.D., McCormack R., Forriol F., Mohtadi N., Pelet S., Desnoyers J., Méthot S., Vehik K., Restrepo A. (2015). BST-CarGel^®^ Treatment Maintains Cartilage Repair Superiority over Microfracture at 5 Years in a Multicenter Randomized Controlled Trial. Cartilage.

[248] Bellich B., D’Agostino I., Semeraro S., Gamini A., Cesàro A. (2016). “The Good, the Bad and the Ugly” of Chitosans. Mar. Drugs.

